# Effects of Using Different Indirect Techniques on the Calculation of Reference Intervals: Observational Study

**DOI:** 10.2196/45651

**Published:** 2023-07-17

**Authors:** Dan Yang, Zihan Su, Runqing Mu, Yingying Diao, Xin Zhang, Yusi Liu, Shuo Wang, Xu Wang, Lei Zhao, Hongyi Wang, Min Zhao

**Affiliations:** 1 National Clinical Research Center for Laboratory Medicine Department of Laboratory Medicine The First Hospital of China Medical University Shenyang China

**Keywords:** comparative study, data transformation, indirect method, outliers, reference interval, clinical decision-making, complete blood count, red blood cells, white blood cells, platelets, laboratory, clinical

## Abstract

**Background:**

Reference intervals (RIs) play an important role in clinical decision-making. However, due to the time, labor, and financial costs involved in establishing RIs using direct means, the use of indirect methods, based on big data previously obtained from clinical laboratories, is getting increasing attention. Different indirect techniques combined with different data transformation methods and outlier removal might cause differences in the calculation of RIs. However, there are few systematic evaluations of this.

**Objective:**

This study used data derived from direct methods as reference standards and evaluated the accuracy of combinations of different data transformation, outlier removal, and indirect techniques in establishing complete blood count (CBC) RIs for large-scale data.

**Methods:**

The CBC data of populations aged ≥18 years undergoing physical examination from January 2010 to December 2011 were retrieved from the First Affiliated Hospital of China Medical University in northern China. After exclusion of repeated individuals, we performed parametric, nonparametric, Hoffmann, Bhattacharya, and truncation points and Kolmogorov–Smirnov distance (kosmic) indirect methods, combined with log or BoxCox transformation, and Reed–Dixon, Tukey, and iterative mean (3SD) outlier removal methods in order to derive the RIs of 8 CBC parameters and compared the results with those directly and previously established. Furthermore, bias ratios (BRs) were calculated to assess which combination of indirect technique, data transformation pattern, and outlier removal method is preferrable.

**Results:**

Raw data showed that the degrees of skewness of the white blood cell (WBC) count, platelet (PLT) count, mean corpuscular hemoglobin (MCH), mean corpuscular hemoglobin concentration (MCHC), and mean corpuscular volume (MCV) were much more obvious than those of other CBC parameters. After log or BoxCox transformation combined with Tukey or iterative mean (3SD) processing, the distribution types of these data were close to Gaussian distribution. Tukey-based outlier removal yielded the maximum number of outliers. The lower-limit bias of WBC (male), PLT (male), hemoglobin (HGB; male), MCH (male/female), and MCV (female) was greater than that of the corresponding upper limit for more than half of 30 indirect methods. Computational indirect choices of CBC parameters for males and females were inconsistent. The RIs of MCHC established by the direct method for females were narrow. For this, the kosmic method was markedly superior, which contrasted with the RI calculation of CBC parameters with high |BR| qualification rates for males. Among the top 10 methodologies for the WBC count, PLT count, HGB, MCV, and MCHC with a high-BR qualification rate among males, the Bhattacharya, Hoffmann, and parametric methods were superior to the other 2 indirect methods.

**Conclusions:**

Compared to results derived by the direct method, outlier removal methods and indirect techniques markedly influence the final RIs, whereas data transformation has negligible effects, except for obviously skewed data. Specifically, the outlier removal efficiency of Tukey and iterative mean (3SD) methods is almost equivalent. Furthermore, the choice of indirect techniques depends more on the characteristics of the studied analyte itself. This study provides scientific evidence for clinical laboratories to use their previous data sets to establish RIs.

## Introduction

Reference intervals (RIs) play an important role in clinical decision-making. For most clinical laboratories, objective RIs are critical benchmarks for identifying healthy and unhealthy populations [1]. However, improper RIs lead to clinical missed and inaccurate diagnoses. Several studies have demonstrated that RIs might be affected by race, age, sex, region, and other factors [2]. Therefore, every laboratory should establish RIs suitable for its specific service populations.

Previous studies have demonstrated that RIs can be established through direct, indirect, and transference methods [2], each of which has particular applicable conditions and relative advantages. Generally, the Clinical and Laboratory Standards Institute (CLSI) endorses direct methods over the other 2 types of methods [2]. However, this gold standard has drawbacks, such as difficulty in recruiting sufficient reference individuals; selection bias in small-sample data; and time, labor, and financial costs [2-4]. For some laboratories that do not have the ability to carry out large-scale epidemiological investigations, it is difficult to establish RIs suitable for their patients through the gold standard. In this case, they need to continue to use the RIs provided by the manufacturer, which may not be appropriate. With the advent of medical big data, the use of indirect methods based on data sets previously obtained from clinical laboratories is promising.

The establishment of RIs using indirect methods roughly involves data acquisition, data cleaning, transformation of skewed data, elimination of outliers or error values, and selection of appropriate statistical methods to calculate the reference limits (RLs) [2,3]. Although indirect methods are obviously more concise in the implementation process compared to direct methods, the selection of an appropriate combination of data transformation, outlier removal, and indirect processes to establish RIs of laboratory analytes with different data distribution characteristics still raises controversies [5].

In 2020, Hickman et al [1] reported that different outlier-processing methods markedly influence the final RIs derived by the direct method. However, whether various outlier removal approaches affect RIs established using indirect methods is unclear. Furthermore, it is crucial to separate the data of “diseased populations” from big data for indirect techniques [6-9]. Many indirect techniques, including parametric and nonparametric approaches [10], the Hoffmann method [11], the Bhattacharya method [12], and truncation points and the Kolmogorov–Smirnov distance (kosmic method) [9], try to cluster data through various mathematical operations in order to obtain “nondiseased populations.” Each of these indirect techniques demonstrates unique characteristics in terms of establishing RIs. Although Ozarda et al [4] previously compared RIs derived using direct and partial indirect methods based on compatible data sets, there has been no systematic evaluation of diverse indirect techniques combined with data transformation approaches and various outlier removals for calculating RLs.

Hence, we systematically and comprehensively explored the effects of various combinations of different statistical techniques used in indirect methods on RI determination and compared the RIs established using different indirect and direct methods. Our results will provide a scientific basis for clinicians to use their own laboratory data to establish RIs suitable for their own service population.

## Methods

The data-processing flowchart of this study is shown in Figure S1 in Multimedia Appendix 1.

### Indirect Methods

#### Data Source and Preliminary Data Cleaning

The probability of illness and interference factors among populations undergoing physical examination is much lower than that of outpatient and emergency patients; thus, in the absence of other clinical diagnostic information, relatively healthy populations undergoing physical examination are more suitable for establishing complete blood count (CBC) RIs using indirect methods. Furthermore, it is too difficult for clinical laboratory researchers to obtain clinical information, which creates a barrier to setting inclusion and exclusion standards for indirect methods. To simulate practical application scenarios, indirect methods derived from physical real-world data have great application and promotion value. A data set of 8 CBC parameters—the red blood cell (RBC) count, hemoglobin (HGB), hematocrit (HCT), the mean corpuscular volume (MCV), mean corpuscular hemoglobin (MCH), the mean corpuscular hemoglobin concentration (MCHC), the platelet (PLT) count, and the white blood cell (WBC) count—for populations aged ≥18 years undergoing physical examination, measured with an XE-2100 hematology analyzer (Sysmex Corp), from January 2010 to December 2011 (24-month period) was retrieved from the laboratory information system in the physical examination center of the First Affiliated Hospital of China Medical University, Shenyang. The relevant instruments and equipment for CBC testing during the study period remained the same. A daily control was performed following the International Council for Standardization in Hematology (ICSH) guidelines.

Next, preliminary data cleaning was performed, as reported by Jones et al [3]. In cases with repeated measures, only the initial data were used in the analysis, since individuals who have more than 1 measurement of analytes are more likely to have diseases. Before data cleaning, the sample size was 42,176. After eliminating 55 (0.13%) repeated measurements, the remaining 42,121 (99.87%) subjects (males: n=24,073, 57.15%) were enrolled in the follow-up analysis.

#### Data Transformation and Treatment of Outliers

The data distribution normality was evaluated by calculating skewness and kurtosis. We used the 2 most commonly used data normalization methods, namely log transformation and BoxCox transformation. After skewed data were transformed through these 2 normalization methods, combined with 3 outlier removal techniques, namely Reed–Dixon, Tukey, and iterative mean (3SD), we obtained 6 processed initial data sets.

#### Statistical Analysis

Data were analyzed using R version 4.1.2 (R Core Team and the R Foundation for Statistical Computing) [13] and Bellview version 1.2.5 [14]. The normality assumptions were first checked using the Kolmogorov–Smirnov test, stratified by sexes. Numerical variables were presented as the median (IQR). Sex differences were compared using the Mann–Whitney *U* test. Next, variations of different analytes with age were illustrated with scatter plots and fitting curves using the cubic spline smoothing function of a generalized additive model.

Subsequent statistical operations were based on the 6 data sets obtained after data transformation and outlier removal, except for nonparametric and kosmic methods. Nonparametric and kosmic methods used the original data set after data transformation and outlier removal, whereas the parametric, Hoffmann, and Bhattacharya methods used the transformed data set after outlier removal to establish RIs.

#### Parametric Method

In this method, we first calculated the mean (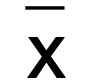) and SD of each data set. Next, transformed RIs were calculated using 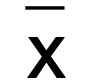 (1.96 SD). Finally, the transformed RIs were inversely converted according to their previous data transformation mode (log or BoxCox transformation) to obtain targeted RIs. This data transformation and inverse transformation were also applied to Hoffmann and Bhattacharya techniques.

#### Nonparametric Method

This method was used per CLSI guidelines. RIs were determined based on the central 95% range of reference values, that is, the lower limits (LLs) and upper limits (ULs) were interpreted as the 2.5th and 97.5th percentiles, respectively.

#### Hoffmann Method

The Hoffmann method mainly requires Gaussian distribution data. We used the data after normalization and outlier removal. This method relies on Q–Q plots and visual inspection of manual intercepts of linear segments [4,15]. A linear segment is extrapolated according to the Hoffmann plot, where the values at the y axis are taken as 2.5% and 97.5% and the range corresponding to the values at the x axis represents the central 95% of the healthy subpopulation, to establish RIs [6]. Analysis was performed using R version 4.1.2 [13], as described by Holmes et al [15].

#### Bhattacharya Method

This is a graphical method requiring Gaussian distribution data. Thus, we used data after normalization and outlier removal [4]. Bellview version 1.2.5 [14] was used to draw log difference plots to illustrate the relationship between log_n+1_ (bin count) – log_n_ (bin count) and the midpoint of the bins [5]. A straight line with a negative slope was constructed by identifying points by eye, and the x intercept and slope were used to calculate the mean and SD, respectively. (Note, the straight line must cover at least 3 points for a valid analysis.) We could input the start and end locations of the bin to determine the straight line, using Bellview version 1.2.5, requiring a Java environment (1.8 or later). These processes were performed manually by the authors, and the basic decision criteria can be found in Bellview instructions.

#### Kosmic Method

The kosmic method primarily uses a truncated power normal distribution family (Gaussian or truncated Gaussian after using BoxCox transformation) to model the proportion of physiological samples. Specifically, this approach minimizes the Kolmogorov–Smirnov distance between an estimated normal distribution and the truncated part of the observed distribution of test results after BoxCox transformation; more specific principles are described by Zierk et al [16]. To simplify these procedures, Zierk et al [16] developed a web-based tool [17], Python bindings, and C++ algorithm implementation, whereas R bindings were explored by Devon Buchanan [18]. In this study, the *tidykosmic* package and the *kosmic* function were applied based on R version 4.1.2.

### The Direct Method

The data set for establishing RIs using the direct method pertained to the data of Han Chinese adults from September 2010 to January 2011 from our previously published study [19] that recruited 4642 healthy individuals from 6 clinical centers in China (Shenyang, Beijing, Shanghai, Guangzhou, Chengdu, and Xi’an). As the data source for the indirect methods was obtained from Shenyang, we used the data previously obtained from Shenyang for the direct method as well. Additionally, we mostly excluded that changes in physiological CBC values are due to variations in social development and nutritional status in the different study periods, because there were no significant differences in the study periods of CBC parameters established using direct (September 2010-January 2011) and indirect (January 2010-December 2011) methods. All subjects fasted for 8-14 hours before sampling. Blood from the cubital vein was used to measure CBC parameters at the First Affiliated Hospital of China Medical University using an XE-2100 hematology analyzer (Sysmex Corp), which was the same as that used for the indirect methods. The precision, carry-over assessment, and linearity of automated hematology analyzers were evaluated according to the ICSH guidelines [20]. Accuracy test results and the acceptable range of bias were determined using standard protocols. Overall, 861 healthy individuals aged 20-79 years (males: n=368, 42.74%; females: n=493, 57.26%) were finally enrolled to establish RIs based on strict criteria; the details are provided elsewhere [19]. Furthermore, related and detailed demographic and general medical information about participants for establishing RIs using the direct method has been previously published as well [19].

#### Assessment of RLs Calculated by Different Methods

This study adopted 2 approaches to evaluate the differences and biases in RLs determined using various methods. We selected RLs determined using the direct method as the reference standard for evaluating the differences among various indirect methods for calculating RIs. One approach was to calculate the relative deviation between RLs. The following formula was used:



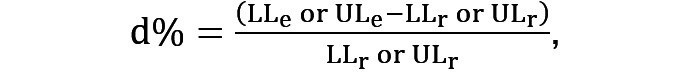



where d% is the difference between the LL/UL for indirect and direct methods, and the LLs and ULs of the RIs to be evaluated and the “reference standard” RIs are LL_e_, UL_e_, LL_r_, and UL_r_, respectively.

The other method was to calculate bias ratios (BRs) [4]. First, the SD of RI (SD_RI_) was calculated as follows:



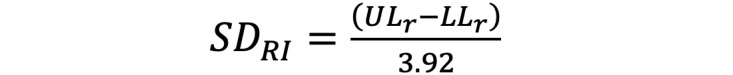



Next, the BRs for LL_e_ and UL_e_ were calculated as follows [21]:



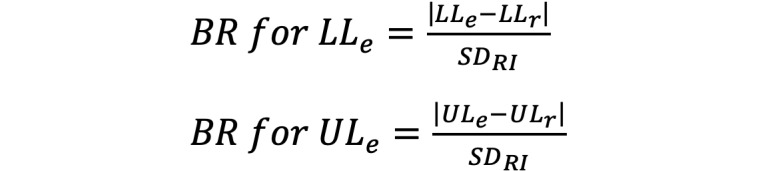



Finally, we regarded BRs<0.375 as the allowable minimum bias [4]. Thus, if the BR was <0.375, RLs derived using indirect methods were not significantly different from the corresponding RLs derived using the direct method.

### Ethical Considerations

This study complies with all the relevant national regulations and institutional policies and the tenets of the Declaration of Helsinki. The study was approved by the Ethics Committee of the First Affiliated Hospital of China Medical University (approval number 2021 442). Informed consent for the direct method was obtained from all individuals included in this study, whereas the indirect method research was exempt from informed consent due to the use of previous laboratory data.

## Results

### Effects of Age and Sex

**Figure 1:**
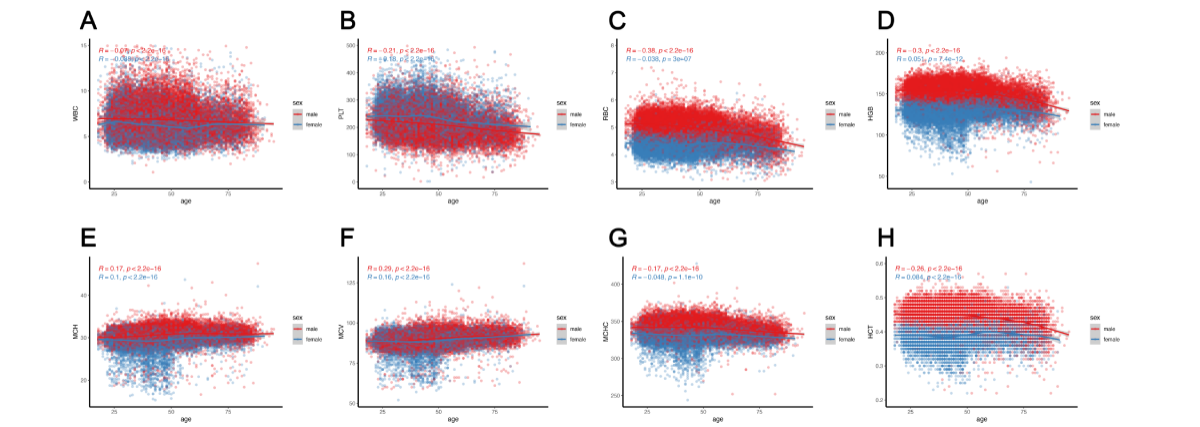
Trends of variation in the levels of CBC parameters with age. (A) WBCs (×10^9^/L), (B) PLTs (×10^9^/L), (C) RBCs (×10^12^/L), (D) HGB (g/L), (E) MCH (pg), (F) MCV (fL), (G) MCHC (g/L), and (H) HCT (L/L). CBC: complete blood count; HCT: hematocrit; HGB: hemoglobin; MCH: mean corpuscular hemoglobin; MCHC: mean corpuscular hemoglobin concentration; MCV: mean corpuscular volume; PLT: platelet; RBC: red blood cell; WBC: white blood cell.

Scatter plots and fitting curves describing the original CBC parameter changes with age for both sexes revealed notable relationships between all the CBC parameters and age (Figure 1). Additionally, physiological levels of 8 CBC parameters revealed significant differences between males and females (Figure 1 and Table 1).

**Table 1:** Sex differences in 8 CBC^a^ parameters after data cleaning.

Analyte	Unit	Male (n=24,073), median (IQR)	Female (n=18,048), median (IQR)	Overall (N=42,121), median (IQR)	*P* value^b^
WBC^c^	×10^9^/L	6.64 (5.68-7.75)	6.09 (5.19-7.11)	6.40 (5.45-7.50)	<.001
RBC^d^	×10^12^/L	4.98 (4.75-5.21)	4.39 (4.19-4.58)	4.72 (4.39-5.05)	<.001
HGB^e^	g/L	152 (146.00-159.00)	130 (124.00-136.00)	143 (131.00-154.00)	<.001
HCT^f^	L/L	0.45 (0.43-0.46)	0.39 (0.38-0.41)	0.42 (0.39-0.45)	<.001
MCV^g^	fL	90 (87.00-92.00)	89 (87.00-92.00)	90 (87.00-92.00)	<.001
MCH^h^	pg	30.6 (29.80-31.50)	29.8 (28.90-30.70)	30.3 (29.40-31.20)	<.001
MCHC^i^	g/L	341 (335.00-347.00)	332 (327-338)	337 (331-344)	<.001
PLT^j^	×10^9^/L	208 (180-239)	230 (199-266)	217 (187-251)	<.001

^a^CBC: complete blood count.

^b^*P* value: The Mann–Whitney *U* test was performed to explore sex differences in CBC parameters (Table S1 in Multimedia Appendix 2), and *P*<.05 was considered statistically significant.

^c^WBC: white blood cell.

^d^RBC: red blood cell.

^e^HGB: hemoglobin.

^f^HCT, hematocrit.

^g^MCV: mean corpuscular volume.

^h^MCH: mean corpuscular hemoglobin.

^i^MCHC: mean corpuscular hemoglobin concentration.

^j^PLT: platelet.

### Characteristics of Data Distribution for CBC Analytes

Tables 2 and 3 illustrate the skewness and kurtosis of raw and processed data for 8 CBC parameters after different data transformation and outlier removal methods were applied. From the results for raw data, we found that data distributions of all the CBC parameters showed different degrees of skew distribution. Among them, the degrees of skewness of the WBC count, PLT count, MCH, MCHC, and MCV were much more obvious than those of other CBC parameters. Additionally, the distribution types of these data were close to the Gaussian distribution after log transformation (Table 2) or BoxCox transformation combined (Table 3) with Tukey or iterative mean (3SD) processing, whereas the Reed–Dixon outlier removal method had no substantial effects on the transformation of data distribution characteristics.

**Table 2:** Skewness and kurtosis of raw data and log transformation–processed data for 8 CBC^a^ parameters after different data transformation and outlier removal methods were applied.

Analyte and sex		Raw data			Reed–Dixon			Tukey			Iterative mean (3SD)	
		Skewness	Kurtosis	Skewness		Kurtosis	Skewness		Kurtosis	Skewness		Kurtosis
**Male**												
	WBC^b^	6.264	243.709	0.158		4.483	0.020		2.732	0.042		2.887
	PLT^c^	0.873	8.840	–1.003		14.253	–0.060		2.742	–0.093		2.937
	RBC^d^	–0.445	5.622	–1.172		11.090	–0.171		2.787	–0.218		2.975
	HGB^e^	–0.752	6.617	–1.530		13.064	–0.106		2.734	–0.202		3.014
	HCT^f^	–0.690	6.484	–1.349		11.595	–0.203		2.717	–0.194		3.034
	MCV^g^	–0.204	8.406	–0.768		10.718	0.090		2.788	0.093		2.902
	MCH^h^	–0.704	11.389	–1.601		17.466	0.060		2.762	0.068		2.981
	MCHC^i^	–0.015	5.041	–0.193		5.897	0.072		2.814	0.088		2.907
**Female**												
	WBC	1.332	11.619	0.085		3.490	0.010		2.737	0.008		2.883
	PLT	0.698	6.632	–0.634		6.622	–0.076		2.765	–0.106		2.925
	RBC	0.064	5.316	–0.565		9.993	–0.056		2.748	–0.066		2.876
	HGB	–1.157	6.853	–1.907		11.560	–0.145		2.757	–0.283		3.080
	HCT	–0.700	5.509	–1.104		6.803	–0.163		2.746	–0.253		3.031
	MCV	–1.614	9.333	–2.115		12.179	–0.107		2.894	–0.199		3.044
	MCH	–2.162	11.334	–2.867		15.873	–0.138		2.822	–0.262		3.131
	MCHC	–1.012	7.856	–1.305		9.112	0.029		2.822	–0.033		3.056

^a^CBC: complete blood count.

^b^WBC: white blood cell.

^c^PLT: platelet.

^d^RBC: red blood cell.

^e^HGB: hemoglobin.

^f^HCT, hematocrit.

^g^MCV: mean corpuscular volume.

^h^MCH: mean corpuscular hemoglobin.

^i^MCHC: mean corpuscular hemoglobin concentration.

**Table 3:** Skewness and kurtosis of raw data and BoxCox transformation–processed data for 8 CBC^a^ parameters after different data transformation and outlier removal methods were applied.

Analyte and sex		Raw data		Reed–Dixon		Tukey		Iterative mean (3SD)	
		Skewness	Kurtosis	Skewness	Kurtosis	Skewness	Kurtosis	Skewness	Kurtosis
**Male**									
	WBC^b^	6.264	243.709	–0.019	4.133	–0.022	2.733	–0.031	2.901
	PLT^c^	0.873	8.840	0.116	5.262	0.066	2.753	0.077	2.949
	RBC^d^	–0.445	5.622	0.034	4.838	0.023	2.760	–0.001	2.948
	HGB^e^	–0.752	6.617	–0.268	4.680	0.008	2.889	0.005	2.998
	HCT^f^	–0.690	6.484	–0.254	4.736	0.059	2.644	0.009	2.989
	MCV^g^	–0.204	8.406	0.120	8.233	0.172	2.778	0.211	2.941
	MCH^h^	–0.704	11.389	–0.001	10.061	0.141	2.762	0.206	2.975
	MCHC^i^	–0.015	5.041	0.026	4.902	0.112	2.819	0.145	2.923
**Female**									
	WBC	1.332	11.619	–0.003	3.424	–0.022	2.740	–0.037	2.888
	PLT	0.698	6.632	0.085	4.578	0.054	2.767	0.074	2.925
	RBC	0.064	5.316	0.064	5.316	0.039	2.731	0.048	2.891
	HGB	–1.157	6.853	–0.608	5.046	0.006	2.801	–0.117	3.113
	HCT	–0.700	5.509	–0.280	4.372	0.035	2.850	–0.136	3.105
	MCV	–1.614	9.333	–1.134	7.729	–0.075	3.040	–0.075	3.040
	MCH	–2.162	11.334	–1.544	8.769	–0.055	2.830	–0.146	3.153
	MCHC	–1.012	7.856	–0.814	6.484	0.037	2.838	0.041	3.109

^a^CBC: complete blood count.

^b^WBC: white blood cell.

^c^PLT: platelet.

^d^RBC: red blood cell.

^e^HGB: hemoglobin.

^f^HCT, hematocrit.

^g^MCV: mean corpuscular volume.

^h^MCH: mean corpuscular hemoglobin.

^i^MCHC: mean corpuscular hemoglobin concentration.

### Comparison of RIs Across 30 Indirect Calculation Methods

**Figure 2:**
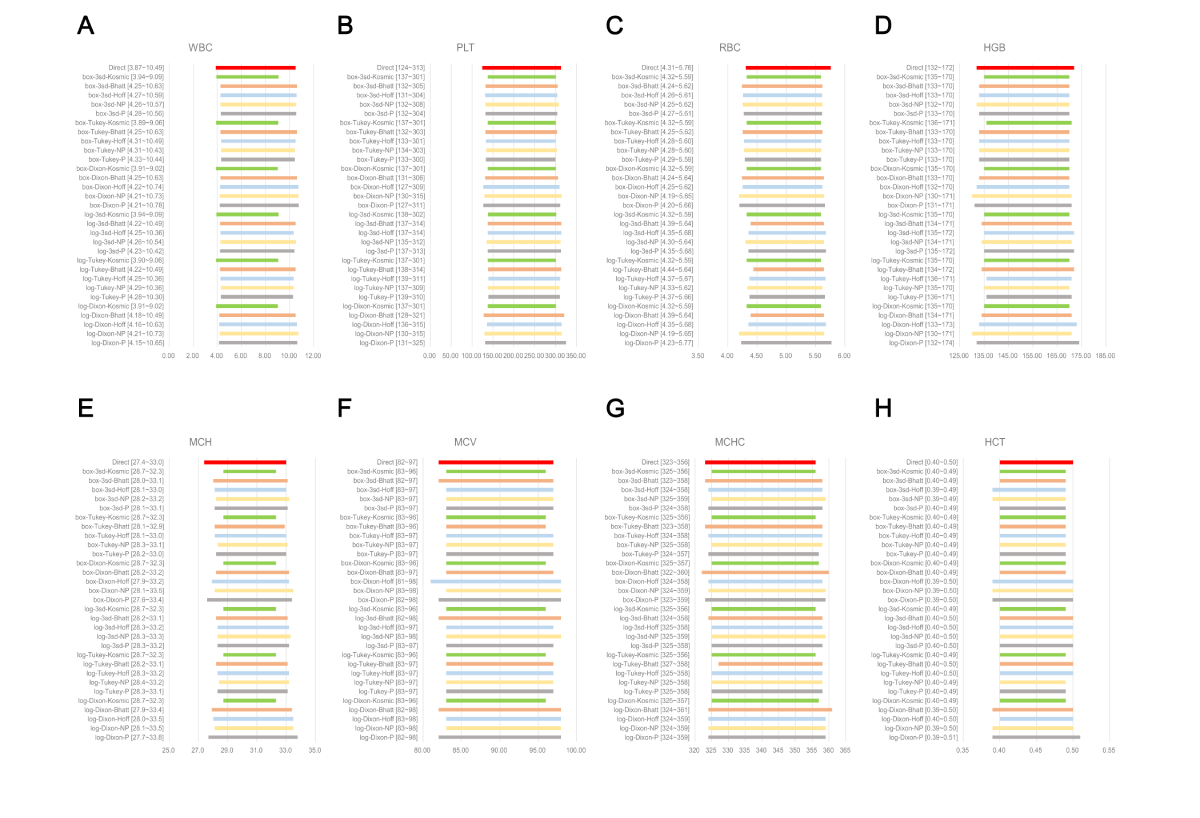
Comparison of RIs for males using 31 calculation methods. (A) WBC (×10^9^/L), (B) PLT (×10^9^/L), (C) RBC (×10^12^/L), (D) HGB (g/L), (E) MCH (pg), (F) MCV (fL), (G) MCHC (g/L), and (H) HCT (L/L). 3SD: mean (3SD) with iteration; Bhatt: Bhattacharya; box: BoxCox transformation; Direct: direct methods; Hoff: Hoffmann; HCT: hematocrit; HGB: hemoglobin; log: log transformation; MCH: mean corpuscular hemoglobin; MCHC: mean corpuscular hemoglobin concentration; MCV: mean corpuscular volume; NP: nonparametric; P: parametric; PLT: platelet; RBC: red blood cell; RI: reference interval; WBC: white blood cell.

**Figure 3:**
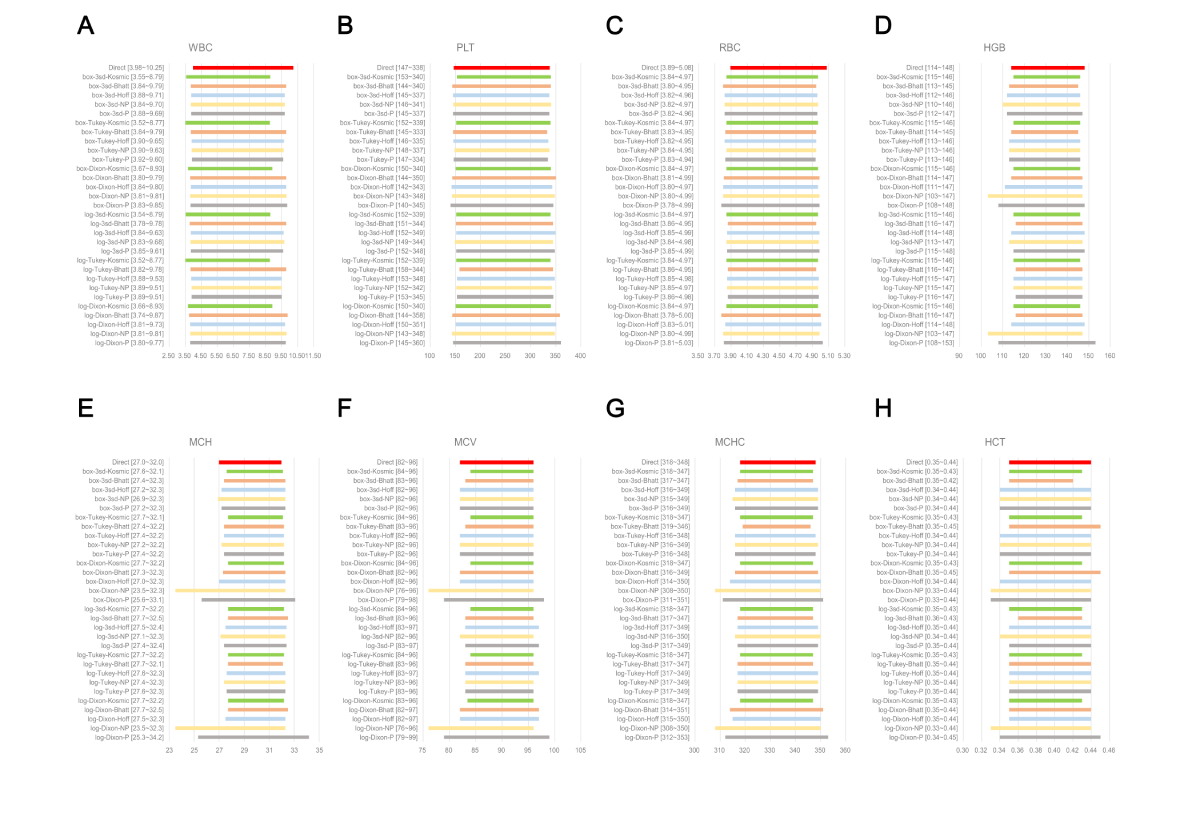
Comparison of RIs for females using 31 calculation methods. (A) WBC (×10^9^/L), (B) PLT (×10^9^/L), (C) RBC (×10^12^/L), (D) HGB (g/L), (E) MCH (pg), (F) MCV (fL), (G) MCHC (g/L), and (H) HCT (L/L). 3SD: mean (3SD) with iteration; Bhatt: Bhattacharya; box: BoxCox transformation; Direct: direct methods; Hoff: Hoffmann; HCT: hematocrit; HGB: hemoglobin; log: log transformation; MCH: mean corpuscular hemoglobin; MCHC: mean corpuscular hemoglobin concentration; MCV: mean corpuscular volume; NP: nonparametric; P: parametric; PLT: platelet; RBC: red blood cell; RI: reference interval; WBC: white blood cell.

The RIs of CBC parameters, established using 30 different indirect methods, are displayed in a bar chart for males (Figure 2) and females (Figure 3). In these charts, we compared the RIs among normalization methods, outlier removal methods, and indirect techniques.

#### Effects of Normalization Methods on RIs

When the outlier removal methods and indirect techniques remained fixed, the UL, LL, or both RLs for all CBC parameters, except the WBC count, among males shifted rightward along the x axis after log transformation compared to BoxCox transformation (Figure 2). Additionally, the normalization method had a greater effect on WBC and PLT RIs than on the RIs of other analytes (Figures 2 and 3).

For WBC data among males, we found that the raw data demonstrated a right-skewed distribution (skewness=6.264, kurtosis=243.709; Tables 2 and 3). After using log transformation combined with 3 different outlier removal methods, the distributions of the processed data all approximated a Gaussian distribution, although the skewness values for all combinations exceeded 0 (Table 2). After using BoxCox transformation combined with the 3 different outlier removal methods, the processed data also approximated a Gaussian distribution but the skewness values showed a nonsignificant, slightly left-skewed distribution (Table 3). Furthermore, only the skewness values for WBC data after log transformation were greater than those after BoxCox transformation among the 8 CBC parameters without considering outlier removal methods (Tables 2 and 3).

For female CBC parameter data, the tendency for change was the same as that for males (Figure 3), and the λ values for both sexes converted by BoxCox transformation are shown in Table S2 in Multimedia Appendix 2. In this study, log transformation was not equivalent to BoxCox transformation, because all the λ values were not equal to 0.

#### Effects of Outlier Removal Methods on RIs

More outliers were eliminated using the Tukey method than using the iterative mean (3SD) and Reed–Dixon methods. For males, the elimination rate using the Tukey method ranged from 1.15% to 3.26% compared to 0.64%-1.77% using the iterative mean (3SD) method and 0% using the Reed–Dixon method (Table S3 in Multimedia Appendix 2). Different outlier removal methods led to different elimination rates in both sexes (Tables S3 and S4 in Multimedia Appendix 2).

With fixed data transformation and indirect techniques, the RIs of analytes were mostly affected by outlier removal methods (Figures 2 and 3). For parametric, nonparametric, and Hoffmann indirect methods, the Reed–Dixon method yielded the widest RI regardless of the data transformation method used (Figure 2). For the WBC count, PLT count, HGB, and RBC count among males, after removing outliers using the Tukey method, the RI width calculated using the Hoffmann, nonparametric, and parametric methods were all narrower than when eliminating extreme values using the Reed–Dixon and iterative mean (3SD) methods. However, there was no constant rule when using the Bhattacharya and kosmic techniques (Figure 2).

### Comparison of RIs Among Indirect Methods

Nonparametric and parametric methods yielded wider RIs of HGB, MCH, MCV, MCHC, and HCT than did the other 3 methods among females (Figure 3).

#### Performance of the Hoffmann Method

The representative Q–Q plots of quantiles of the Gaussian distribution of CBC parameters in males and females, after using different data transformation and outlier removal methods, are displayed in Figures S2 and S3 in Multimedia Appendix 1, respectively. The data included in the analysis of CBC parameters after applying Tukey and iterative mean (3SD) methods formed a substantial straight line in the middle of the chart (Figures S2 and S3 in Multimedia Appendix 1). However, the Reed–Dixon method yielded the narrowest linear range for all CBC parameters (Figures S2 and S3 in Multimedia Appendix 1). Different data transformation modes had little effect on the linear range (Figures S2 and S3 in Multimedia Appendix 1). The RI width obtained with the Reed–Dixon method was significantly wider than that obtained with the other 2 outlier removal methods when calculating RIs using the Hoffmann method (Figures 1 and 2). Furthermore, the representative Q–Q plots for MCV and HCT illustrated that the linear relationship is not ideal. Table S5 in Multimedia Appendix 2 shows details of the start and end points of the Hoffmann method.

#### Performance of the Bhattacharya Method

Gaussian populations for establishing RIs were selected by identifying points that formed a straight line with a negative slope (Figures S4-S19 in Multimedia Appendix 1). A least-square regression model was constructed to determine the mean and SD from the x intercept and slope of the straight line, respectively (Figures S4-S19 in Multimedia Appendix 1). From log difference plots, we found it difficult to draw a straight line to cover at least 4 points for transformed CBC parameter data after using the Reed–Dixon method to remove outliers (Figures S4-S19 in Multimedia Appendix 1). However, straight lines were much easier to mark for transformed data after using Tukey and iterative mean (3SD) methods (Figures S4-S19 in Multimedia Appendix 1). Additionally, not all transformed data had small fluctuations on the log difference plots after using Tukey and iterative mean (3SD) methods to remove outliers, such as the data for HGB, MCV, MCHC, and HCT among both sexes (Figures S7, S9-S11, S15, and S17-S19 in Multimedia Appendix 1). Table S6 in Multimedia Appendix 2 shows details of the start and end points of the Bhattacharya method. The operations can be repeated based on the decision criteria (ie, start and end points) recorded in Table S6 in Multimedia Appendix 2.

#### Performance of the Kosmic Method

The estimated distributions of physiological test results and RIs are shown in Figures S20 and S21 in Multimedia Appendix 1. Compared with the influence of outlier removal methods on the kosmic method, the data transformation approaches had little effect (Figure 2). From the frequency distribution histograms for males, the WBC and PLT distributions showed a slight rightward deviation after data processing, while the other parameters had a normal or approximately Gaussian distribution (Figure S20 in Multimedia Appendix 1). For PLT RIs among males and females, outlier removal methods had clear effects when using the kosmic method after log and BoxCox transformations (Figure 3). For the RBC count, HGB, MCH, MCV, and MCHC, the results obtained using the kosmic method were more stable than those obtained using the other 4 indirect methods, and they were not affected by data transformation and outlier removal methods (Figure 3).

### Comparison of RIs Between Direct and Different Indirect Methods

**Figure 4:**
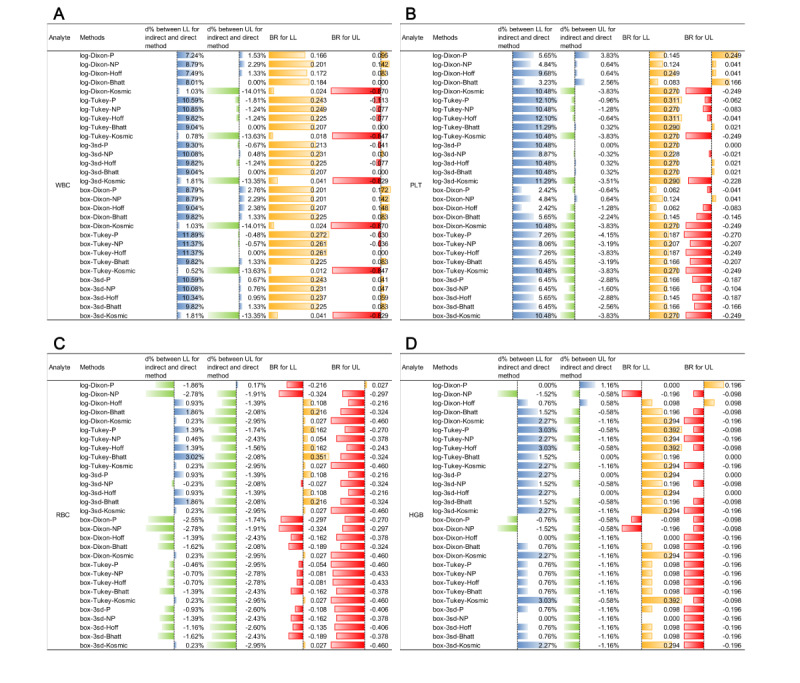
Comparison of RIs for (A) WBC count, (B) PLT count, (C) RBC, and (D) HGB for males using 31 indirect methods with calculation of bias at RLs. (A) WBC (×10^9^/L), (B) PLT (×10^9^/L), (C) RBC (×10^12^/L), and (D) HGB (g/L). 3SD: mean (3SD) with iteration; Bhatt: Bhattacharya; box: BoxCox transformation; Hoff: Hoffmann; BR: bias ratio; d% between LL: relative deviation of lower RL between indirect and direct methods; d% between UL: relative deviation of upper RL between indirect and direct methods; HGB: hemoglobin; LL: lower limit; log: log transformation; PLT: platelet; RBC: red blood cell; RI: reference interval; RL: reference limit; UL: upper limit; WBC: white blood cell.

**Figure 5:**
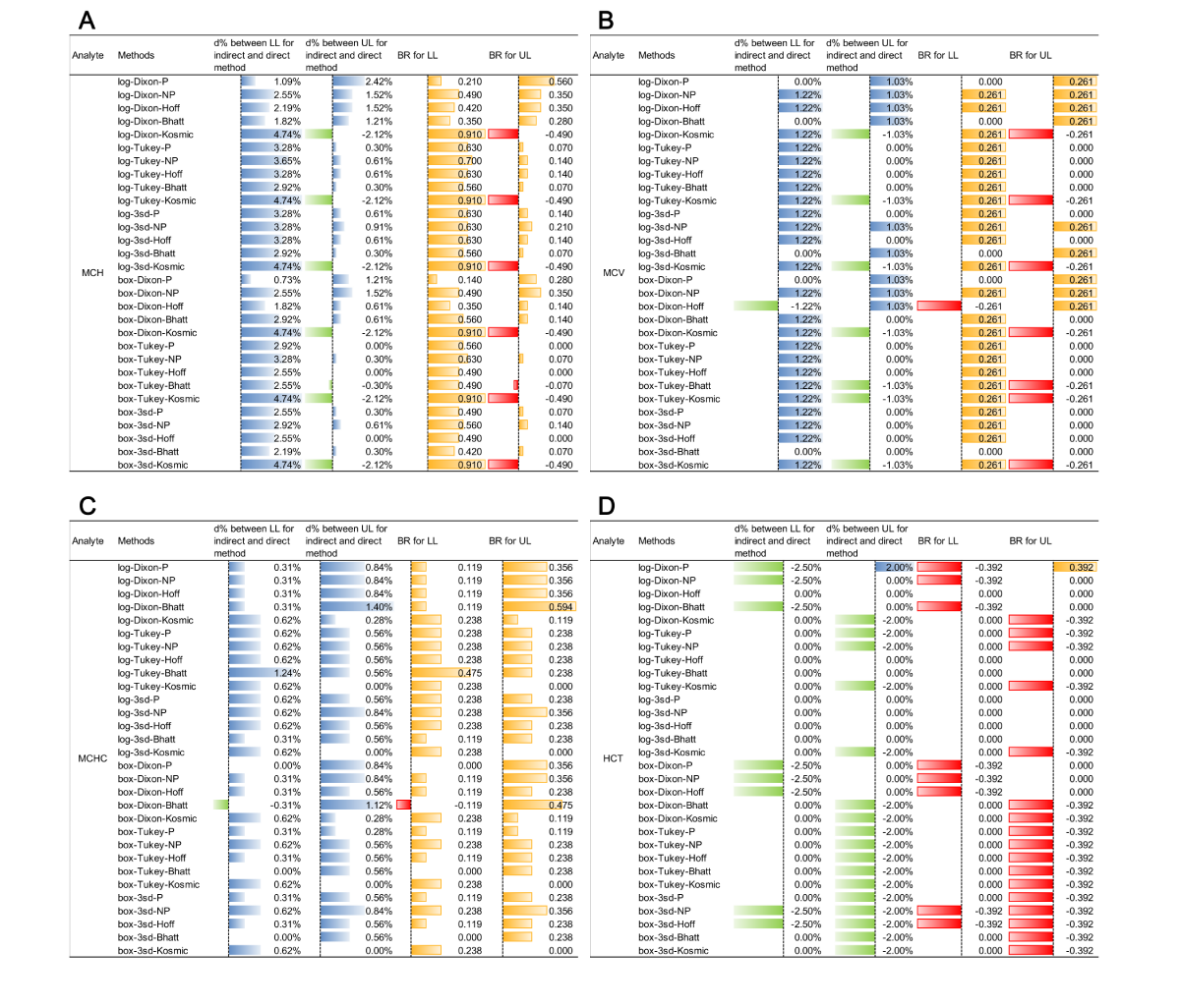
Comparison of RIs of (A) MCH, (B) MCV, (C) MCHC, and (D) HCT for males using 31 indirect methods with calculation of bias at RLs. (A) MCH (pg), (B) MCV (fL), (C) MCHC (g/L), and (D) HCT (L/L). 3SD: mean (3SD) with iteration; Bhatt: Bhattacharya; box: BoxCox transformation; HCT: hematocrit; Hoff: Hoffmann; BR: bias ratio; d% between LL: relative deviation of lower RL between indirect and direct methods; d% between UL: relative deviation of upper RL between indirect and direct methods; LL: lower limit; log: log transformation; MCH: mean corpuscular hemoglobin; MCHC: mean corpuscular hemoglobin concentration; MCV: mean corpuscular volume; RI: reference interval; RL: reference limit; UL: upper limit.

Figures 4 and 5 and Figures S22 and S23 in Multimedia Appendix 1 present a comparison of the biases of RIs calculated using indirect methods with those established using the direct method in males and females, respectively. Compared with the RIs of CBC parameters calculated using the direct method, the LL bias of WBC (male), PLT (male), HGB (male), MCH (male/female), and MCV (female) were greater than that of the corresponding UL for more than half of the 30 indirect methods (Figures 4 and 5).

First, the |BR| values of the LL and UL were sorted in ascending order. Table 4 illustrates the proportion of |BRLL or BRUL|<0.375 and the intersection of the top 10 methodologies (if some methods shared the 10th place, all methods sharing the 10th rank were involved in drawing the Venn diagram for |BRLL| and |BRUL|) corresponding to the ordered |BR| values. Biases of the LL or UL (or both) of some indicators were large. Among the 30 indirect calculation methods, the qualified rate (proportion of |BR|<0.375) of the |BR| for many analytes was above 0 (Table 4).

We also found the following:

- Only a large bias of LLs (ratio of |BRLL|<85%): HGB (female), MCV (female), MCH (female), and MCHC (female)
- Only a large bias of ULs (ratio of |BRUL|<85%): WBC (male/female) and RBC (male/female)
- A large bias of both limits (ratios of |BRLL| and |BRUL| both<85%): MCH (male) and HCT (male/female)

See Figures 4 and 5, Figures S22 and S23 in Multimedia Appendix 1, and Table 4. Although the qualified rate of |BR| for the RLs for MCH and HCT was a little lower, the fluctuation range for |d%| of the LLs and ULs for MCH in both sexes was just 0.73%-4.74% and 0.31%-12.96%, respectively (Figure 5 and Figure S23 in Multimedia Appendix 1). In particular, the range of |d%| of the LLs in male HCT could even be between 0% and 2.50% (Figure 5D).

Data transformation would slightly affect the comparison between the direct and indirect methods. When we compared the BR of RLs for males and females, we found that log (43 times) and BoxCox (41 times) transformations were similar in the top 10 methodologies (Table 4). Furthermore, for the selection of outlier processing with indirect methods, we found that Tukey (30 times) and mean (3SD; 35 times) methods had similar effects, whereas the existence of the Reed–Dixon method (19 times) was usually accompanied with the use of Hoffmann, Bhattacharya, and kosmic methods (Table 4).

For the selection of indirect techniques, computational choices of CBC parameters for males and females were inconsistent. The RIs of MCHC established using the direct method for females were narrow. For this, the kosmic method was markedly superior (Table 4). This contrasted with the RI calculation for CBC parameters with high |BR| qualification rates for males. Among the top 10 methodologies for indicators with a high |BR| qualification rate (WBC count, PLT count, HGB, MCV, and MCHC) among males, the Bhattacharya method appeared 5 times, both parametric and Hoffmann methods appeared 4 times, and the nonparametric method appeared 2 times, whereas the kosmic method did not feature at all (Table 4).

**Table 4:** Ratio of the |BR|^a^ for lower and upper RLs^b^ of <0.375, based on 30 indirect methods and the intersection of the top 10 combinations of data transformation, outlier removal, and indirect techniques for descending |BRLL|^c^ or |BRUL|^d^.

Analyte and sex		Ratio of |BRLL|<0.375 (%)	Ratio of |BRUL|<0.375 (%)	Top methods
**Male**				
	WBC^e^	100.00	80.00	log^f^-Dixon-Bhatt^g^
	PLT^h^	100.00	100.00	box^i^-Dixon-P^j^, log-Dixon-NP^k^, box-Dixon-NP
	RBC^l^	100.00	46.67	log-3SD^m^-NP
	HGB^n^	90.00	100.00	log-Dixon-Hoff^o^, box-Dixon-P
	MCH^p^	13.33	76.67	box-3SD-Bhatt, box-Tukey-Hoff, box-Tukey-Bhatt, box-3SD-P, box-3SD-Hoff
	MCV^q^	100.00	100.00	box-3SD-Bhatt
	MCHC^r^	96.67	93.33	box-Tukey-Bhatt, box-3SD-Bhatt, log-3SD-Bhatt, box-Dixon-Hoff, box-Tukey-P, box-Tukey-Hoff, box-3SD-P, box-3SD-Hoff
	HCT^s^	73.33	40.00	log-Dixon-Hoff, log-Tukey-Hoff, log-Tukey-Bhatt, log-3SD-P, log-3SD-NP, log-3SD-Hoff, log-3SD-Bhatt
**Female**				
	WBC	100.00	53.33	box-Dixon-Hoff, box-Tukey-Bhatt, box-3SD-Bhatt
	PLT	100.00	93.33	box-Tukey-NP, box-3SD-P, box-3SD-Hoff, log-Dixon-kosmic, box-Dixon-kosmic, box-3SD-Bhatt
	RBC	100.00	70.00	log-Tukey-P, log-Tukey-Hoff, log-3SD-P, log-3SD-Hoff, log-3SD-NP
	HGB	83.33	96.67	log-Dixon-Hoff, log-3SD-Hoff, box-Dixon-Bhatt, log-Tukey-NP, log-Tukey-Hoff, log-3SD-P, log-3SD-NP
	MCH	43.33	86.67	box-Tukey-NP, box-Tukey-P, box-Tukey-Hoff, box-Tukey-Bhatt
	MCV	66.6	93.33	log-3SD-NP, box-Dixon-Hoff, box-Dixon-Bhatt, box-Tukey-P, box-Tukey-NP, box-Tukey-Hoff, box-3SD-P, box-3SD-NP, box-3SD-Hoff
	MCHC	73.33	90.00	log-Dixon-kosmic, log-Tukey-kosmic, log-3SD-kosmic, box-Dixon-kosmic, box-Tukey-kosmic, box-3SD-kosmic, log-Tukey-P, log-Tukey-NP, log-Tukey-Hoff, log-Tukey-Bhatt, log-3SD-P, log-3SD-Hoff, log-3SD-Bhatt, box-3SD-Bhatt
	HCT	56.67	63.33	log-Dixon-Hoff, log-Dixon-Bhatt, log-Tukey-P, log-Tukey-NP, log-Tukey-Hoff, log-Tukey-Bhatt, log-3SD-P, log-3SD-Hoff

^a^BR: bias ratio.

^b^RL: reference limit.

^c^LL: lower limit.

^d^UL: upper limit.

^e^WBC: white blood cell.

^f^log: log transformation.

^g^Bhatt: Bhattacharya method.

^h^PLT: platelet.

^i^box: BoxCox transformation.

^j^P: parametric.

^k^NP: nonparametric.

^l^RBC: red blood cell.

^m^3SD: iterative mean (3SD).

^n^HGB: hemoglobin.

^o^Hoff: Hoffmann method.

^p^MCH: mean corpuscular hemoglobin.

^q^MCV: mean corpuscular volume.

^r^MCHC: mean corpuscular hemoglobin concentration.

^s^HCT: hematocrit.

## Discussion

### Principal Findings

To the best of our knowledge, this is the first study to comprehensively evaluate the effects of combinations of different data transformation, outlier removal, and indirect techniques on establishing RIs for large-scale data. For most laboratories that are unable to carry out direct method research, this study provides a scientific and reasonable basis for their use of previous laboratory data sets to establish RIs using indirect methods. Moreover, we used data derived from the direct method as reference standards. We found that for data with different distribution characteristics, outlier removal method and indirect technique use markedly influenced the final RIs, whereas data transformation had negligible effects except for obviously skewed data.

### Strengths

There are several strengths of this study. First, the samples for direct and indirect methods were derived from the same study area and were tested in the same laboratory using the same instrumentation as well. This eliminated many confounding factors for the subsequent combined evaluation of indirect methods. Second, only harmonization of results requires multicenter studies in a given region or country. Thus, this single-center study will encourage laboratory researchers to attempt to establish RIs suitable for their own laboratories. Third, to mostly eliminate the interference of “diseased populations,” we selected subjects undergoing physical examination rather than outpatient or inpatient individuals. Furthermore, to ensure that the “reference population” excluded as much as possible individuals who were sick and considering that individuals who have repeated measurement data are more likely to have abnormalities or diseases, we included only the earliest visit records [22]. We only processed the repeated measurement data in the data-cleaning step, although this step had no substantive impact on the overall data distribution.

For CBC parameters, we found significant differences between the sexes and minor variation among age groups based on the data used for indirect methods. This phenomenon is essentially consistent with Takami et al [22], who reported RIs of the RBC count for healthy adults in Japan and calculated the SD ratio to measure the magnitude of sex differences. They found that sex differences existed in HGB, HCT, RBC count, and MCHC, as the SD ratios of these 4 analytes exceeded 0.3 [22]. Additionally, South African studies have shown that there are sex differences for the WBC and PLT counts as well [23]. Haeckel et al [24] once explored the importance of correct stratifications when comparing directly and indirectly estimated RIs. They suggested that the requirement for stratification should not been neglected during RI determination, with the main variables affecting RIs being sex and age. Considering these viewpoints, at the beginning of this study, confounding factors (ie, sex and age) were explored, and we found that significant sex differences exist in CBC parameters. Therefore, subsequent calculations were stratified by sex.

When exploring the impact of different data transformation methods used with indirect techniques on calculating RIs, there were slight variances between log and BoxCox transformations for different types of data distribution. In most cases, the absolute skewness values for BoxCox transformation were lower than those for log transformation, which means that the effect of BoxCox transformation is better than that of log transformation when λ is not equal to 0, like in this study. For some skewed distribution data, BoxCox transformation might result in overcorrection. For example, raw WBC data in males showed a right-skewed distribution. After log transformation, the data could approximate a Gaussian distribution, although the skewness value still exceeded 0. In contrast to the effect of log transformation, the skewness value of the transformed data was less than 0 after BoxCox transformation, implying correction to a left-skewed distribution. Consequently, raw WBC data for males shifted rightward on the abscissa after BoxCox transformation, which explains the horizontal rightward shift of RIs calculated after BoxCox transformation. Data transformation may bring the pathological population closer to the healthy population, thereby making it more difficult to separate them. However, in this study, compared with the impact of outlier processing and indirect technology on the results, data transformation had a slight impact on the final results.

There have been many studies on the significant effects of the chosen outlier removal method on RIs [1,25,26]. Eggers et al [26] and Hickman et al [25] used direct sampling techniques and evaluated the influence of different outlier-processing methods on the 99th percentile of cardiac troponin values. They found that the UL of the RI is sensitive to the choice of the outlier removal method. Hickman et al [25] stated that the RIs of all analytes would potentially be affected by the outlier removal method. Our study used the indirect sampling technique to calculate the RIs of CBC parameters. These analytes differed from cardiac troponin with regard to the data distribution type. The former often approximates a Gaussian distribution, while the latter shows an obviously skewed distribution. However, in this study, Reed–Dixon, Tukey, and iterative mean (3SD) methods had significant effects on the RIs of CBC parameters as well. Hickman et al [1] reported that various outlier removal methods could markedly change RIs. In their study, the Tukey method more often yielded narrower RIs [1]. The outlier removal efficiency of the Tukey method was similar to that of the iterative mean (3SD) method, consistent with our study. The RI often covers 95% of the healthy population. Furthermore, the Tukey method eliminates about 1% [1]. Therefore, when Tukey method–processed data are used to calculate 95% of the RI, it actually calculates 94% of the RI. This is the fundamental reason for the narrower RI obtained with the Tukey method. When establishing RIs using the direct method, the populations used to calculate RIs are unlikely to be contaminated by “diseased populations,” because the a priori–set exclusion criteria are strict. In the absence of clear evidence that the individual value is abnormal, each participant’s value confirmed by exclusion criteria should be retained in the subsequent calculation. Therefore, the selection of the outlier removal method for the direct technique is inclined to be conservative. However, when using indirect methods on big data, due to the difficulty of applying strict screening criteria, the applicability of conservative outlier removal methods, such as the Reed–Dixon method, in this type of research is poor.

When we explored the influence of different indirect techniques on RIs, we found that data transformation and outlier removal methods had different effects on the results of subsequent parametric, nonparametric, Hoffmann, and Bhattacharya methods. Outlier removal had a larger effect than data transformation on indirect techniques. There was a recent publication examining 8 different indirect methods for RI derivation [27]. The authors conducted 8 methods to simulate 4 different scenarios of mixed populations and distributions. They found that the results derived by the kosmic method in most simulation scenarios are within the allowable error range, and a high proportion of pathological subgroups significantly reduce the performance of the indirect method [27]. However, the median absolute deviation (MAD) and double median absolute deviation (dMAD) involved in that paper study are not discussed in our study. In addition, our research used real-world data and selected results calculated using the direct method as a reference to evaluate the accuracy of commonly used indirect methods, including the parametric, Hoffmann, and Bhattacharya methods that have not been evaluated by Tan et al [27]. The similarity of these 2 studies is that no matter which data transformation and outlier-processing methods are used, the calculation results of the kosmic method are the most stable ones, since they are minimally affected by these data-preprocessing measures. In contrast to parametric and nonparametric methods, even for big data, after data cleaning and outlier removal, the kosmic method recognized that both pathological and nonpathological values were present. The core of this method is that the estimation of the proportion of physiological samples can be modeled with a parametric distribution and minimizes the Kolmogorov–Smirnov distance between a hypothetical Gaussian distribution and the observed distribution of test results after BoxCox transformation [9,16]. For the WBC count, the upper RL estimated by the kosmic method was quite different from that calculated using the direct method. This could be due to the limitations of the kosmic method itself [16] or because among the population undergoing physiological examination, the probability of abnormal WBC test results is significantly higher than that of other CBC parameters. If there is much overlap between abnormal and physiological test results (ie, the abnormal test results are more likely to be close to the UL or LL), the BR between indirect and direct methods increases. Since Hoffmann and Bhattacharya methods depend on linearity [12,28], they are both highly sensitive to data normality and the presence of outliers. Although both methods use the middata for calculation, too many values were retained to establish a linear equation in this study. The reason for this phenomenon is that raw distributions of most CBC parameters approximated a Gaussian distribution and fewer values with a negative impact on linearity were eliminated at both ends of the line. Furthermore, a different bin size was optimized, and it was difficult for different authors involved in the subjective selection of the straight line to retain consistency. However, we followed the same principle in the process of value selection and recorded the location of the bin to ensure the repeatability of the study as much as possible. When comparing the effects of combinations of indirect techniques, data transformation, and outlier removal methods on RIs with reference to the direct method RIs, the width of the RIs established using the direct method was wider (larger intraindividual variation) and those calculated by Hoffmann, nonparametric, Bhattacharya, and parametric methods were closer to those calculated using the direct method. For data with smaller intraindividual variation, the width of the RIs determined using the direct method was narrower; the kosmic method that excluded a large number of pathological values seemed to be markedly more applicable.

### Limitations

This study has a few limitations. First, it is essential to consider the requirement of correct stratifications when comparing directly and indirectly estimated RIs. Both direct and indirect methods lead to erroneous RIs if stratification is performed for unknown variables. However, this study explored 2 important factors affecting RIs, namely sex and age, in order to eliminate the interference of these factors in the comparison of results as much as possible. Second, the 5 indirect methods used in this study were all based on unimodal data. Thus, the calculation was simply based on the distribution rule of the data itself, which inevitably causes contamination of the “reference population” with the “diseased population” to the fullest extent. The exclusion of “diseased populations” using statistical tools is obviously insufficient. Therefore, in the future, we will obtain as much multimodal data as possible during the data-cleaning phase in order to construct an unsupervised classification model to ensure that the included “reference population” is healthy. Next, this study selected results derived using the direct method as a reference. However, the direct sampling of the population may not be truly representative and is always subject to methodological bias and variability. To ensure the comparability of the results of the indirect and direct methods, this study selected the same research period, the same detecting system, and the same sampling location to avoid the risk of bias as much as possible. In addition, the direct method was used as a measurement standard of the indirect method results, which was also applied by Ozarda et al [4]. We also advocate that in the process of establishing RIs, a fusion of direct and indirect methods can be applied, and the screening criteria of the direct method can be applied to the data sources of the indirect methods so as to obtain more representative RIs. Additionally, this study only compared the RIs of CBC parameters established using direct and indirect methods. However, for analytes such as alanine aminotransferase, RIs are quite different compared to CBC parameters. More interfering factors, such as smoking, drinking, exercise, and eating habits, might have significant effects on such analytes. Thus, whether our conclusions are applicable remains to be further discussed in the future. Finally, this study only covered 5 commonly used indirect methods, while many other complicated machine learning approaches exist. In the future, we will evaluate these emerging methods to provide much more comprehensive guidance on indirect methods for establishing accurate RIs.

### Conclusion

In summary, this comparative study investigated indirect methods for establishing RIs, and the results provide a valuable scientific basis for method selection by laboratory clinicians. Compared to the results of the direct method, the selection of outlier removal methods and indirect techniques markedly affects the final RIs, whereas the effects of data transformation are negligible except for obviously skewed data. Specifically, the outlier removal efficiency of Tukey and iterative mean (3SD) methods is almost equivalent. Furthermore, the choice of indirect techniques depends more on the characteristics of the studied analyte itself. Use of the kosmic method to establish RIs of analytes with large intraindividual variations is not recommended. Furthermore, each laboratory should develop its own RIs under the applicable conditions. This study provides a new scientific basis for establishing RIs for laboratories at any level. In the future, we will explore more efficient indirect techniques based on multimodal data. Evaluation of the accuracy and applicability of RIs estimated using indirect methods is also needed, particularly in the absence of direct data as a reference.

## Appendix

Multimedia Appendix 1 Supplementary figures.

Multimedia Appendix 2 Supplementary tables.

## Abbreviations

BR: bias ratio
CBC: complete blood count
CLSI: Clinical and Laboratory Standards Institute
HCT: hematocrit
HGB: hemoglobin
ICSH: International Council for Standardization in Hematology
LL: lower limit
MCH: mean corpuscular hemoglobin
MCHC: mean corpuscular hemoglobin concentration
MCV: mean corpuscular volume
PLT: platelet
RBC: red blood cell
RI: reference interval
RL: reference limit
UL: upper limit
WBC: white blood cell

## Data Availability

The data sets used and analyzed during the study are available from the corresponding author upon reasonable request.
