# Supplementary material for: Effects of Using Different Indirect Techniques on the Calculation of Reference Intervals: Observational Study
Source: J Med Internet Res. 2023 Jul 17;25:e45651. doi: 10.2196/45651 (PMC10390978; doi:10.2196/45651)
Supplement: Multimedia Appendix 1 [file jmir_v25i1e45651_app1.docx]

**Supplementary materials**

**Statistical consideration of indirect techniques combined with different data transformation methods and outlier removal causes substantial differences in calculation of reference intervals**

**Short title:** indirect statistical considerations for reference intervals

**Authors:**

Dan Yang ^1, 2^, Zihan Su ^1, 2^, Runqing Mu ^1, 2^, Yingying Diao ^2^, Xin Zhang ^1, 2^, Yusi Liu ^2^, Shuo Wang  ^2^ , Xu Wang ^1^, Lei Zhao ^1, 2^, Hongyi Wang ^1, 2^, Min Zhao *^1, 2^

**Affiliations of all authors:**

1. National Clinical Research Center for Laboratory Medicine, The First Hospital of China Medical University, Shenyang, P. R. China

2. Department of Laboratory Medicine, The First Hospital of China Medical University, Shenyang, P. R. China

**Correspondence to**

*1 Min Zhao, Ph. D

National Clinical Research Center for Laboratory Medicine,

Department of Laboratory Medicine,

The First Hospital of China Medical University,

Shenyang, P. R. China

E-mail: [minzhao@cmu.edu.cn](mailto:minzhao@cmu.edu.cn)

Telephone: +86 13898169877

**Suppl Fig. 1 Flow chart for data processing**

**
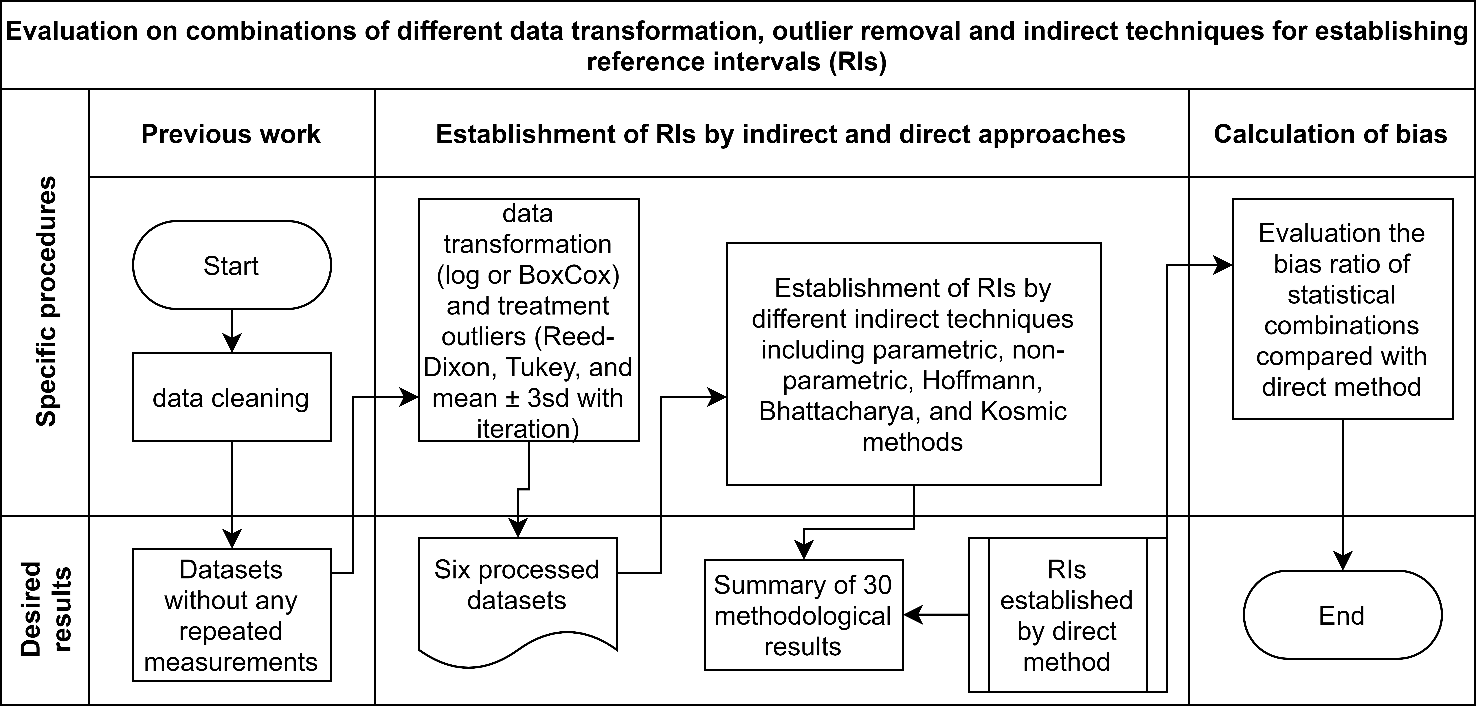
**

**Suppl Fig. 2 The representative Q-Q plots of quantiles of the normal distribution of CBC parameters after different data transformation and outlier removal methods for males**

A-H represent A: WBC (×10^9^/L); B: PLT (×10^9^/L); C: RBC (×10^12^/L); D: HGB (g/L); E: MCH (pg); F: MCV (fL); G: MCHC (g/L); H: HCT (L/L), respectively. 1-6 represent 1: log+Dixon; 2: log+Tukey; 3: log+mean±3sd; 4: BoxCox+Dixon; 5: BoxCox+Tukey; 6: BoxCox+mean±3sd, respectively.

**
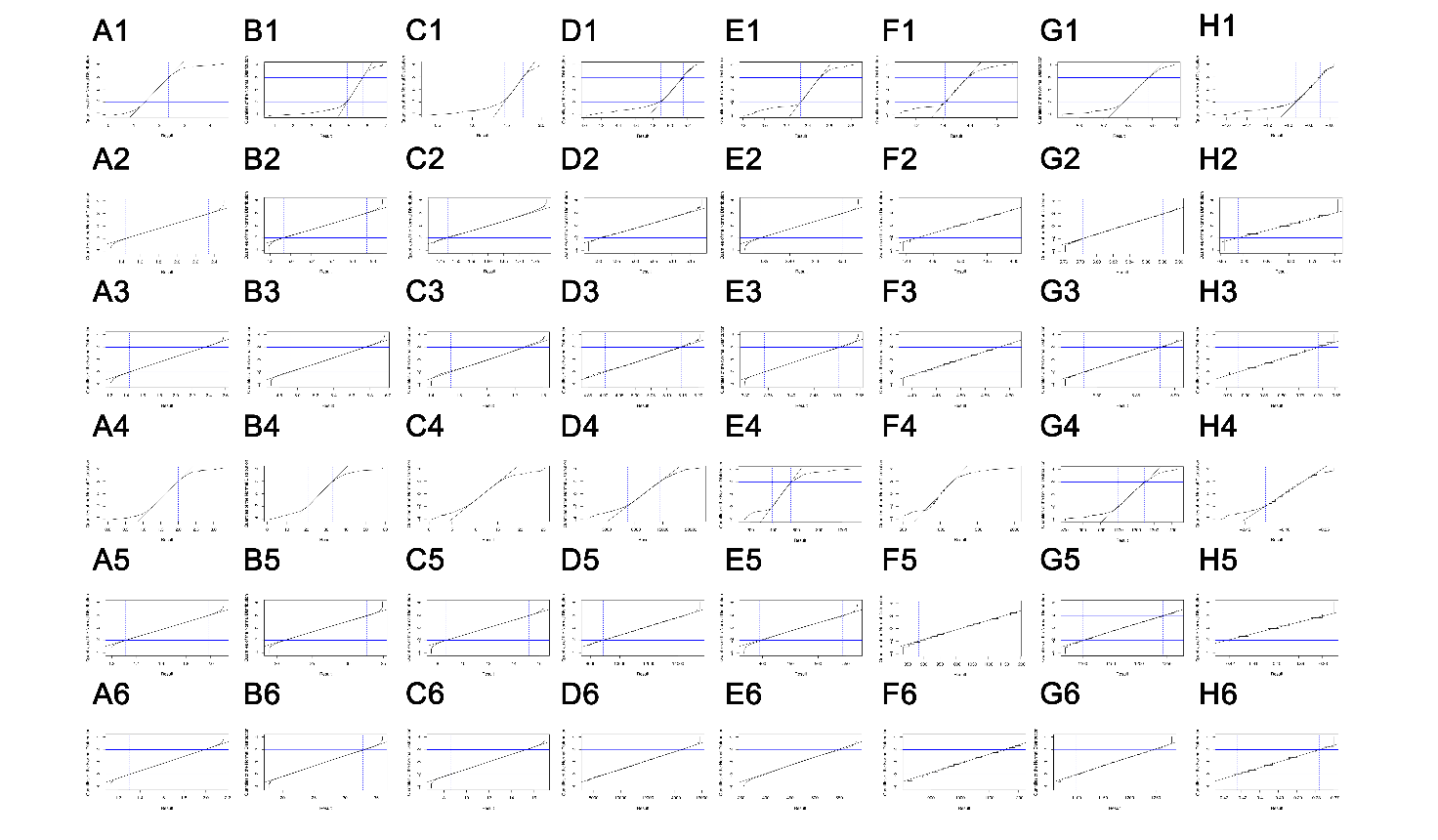
**

**Suppl Fig. 3 The representative Q-Q plots of quantiles of the normal distribution of CBC parameters after different data transformation and outlier removal methods for females**

A-H represent A: WBC (×10^9^/L); B: PLT (×10^9^/L); C: RBC (×10^12^/L); D: HGB (g/L); E: MCH (pg); F: MCV (fL); G: MCHC (g/L); H: HCT (L/L), respectively. 1-6 represent 1: log+Dixon; 2: log+Tukey; 3: log+mean±3sd; 4: BoxCox+Dixon; 5: BoxCox+Tukey; 6: BoxCox+mean±3sd, respectively.

**
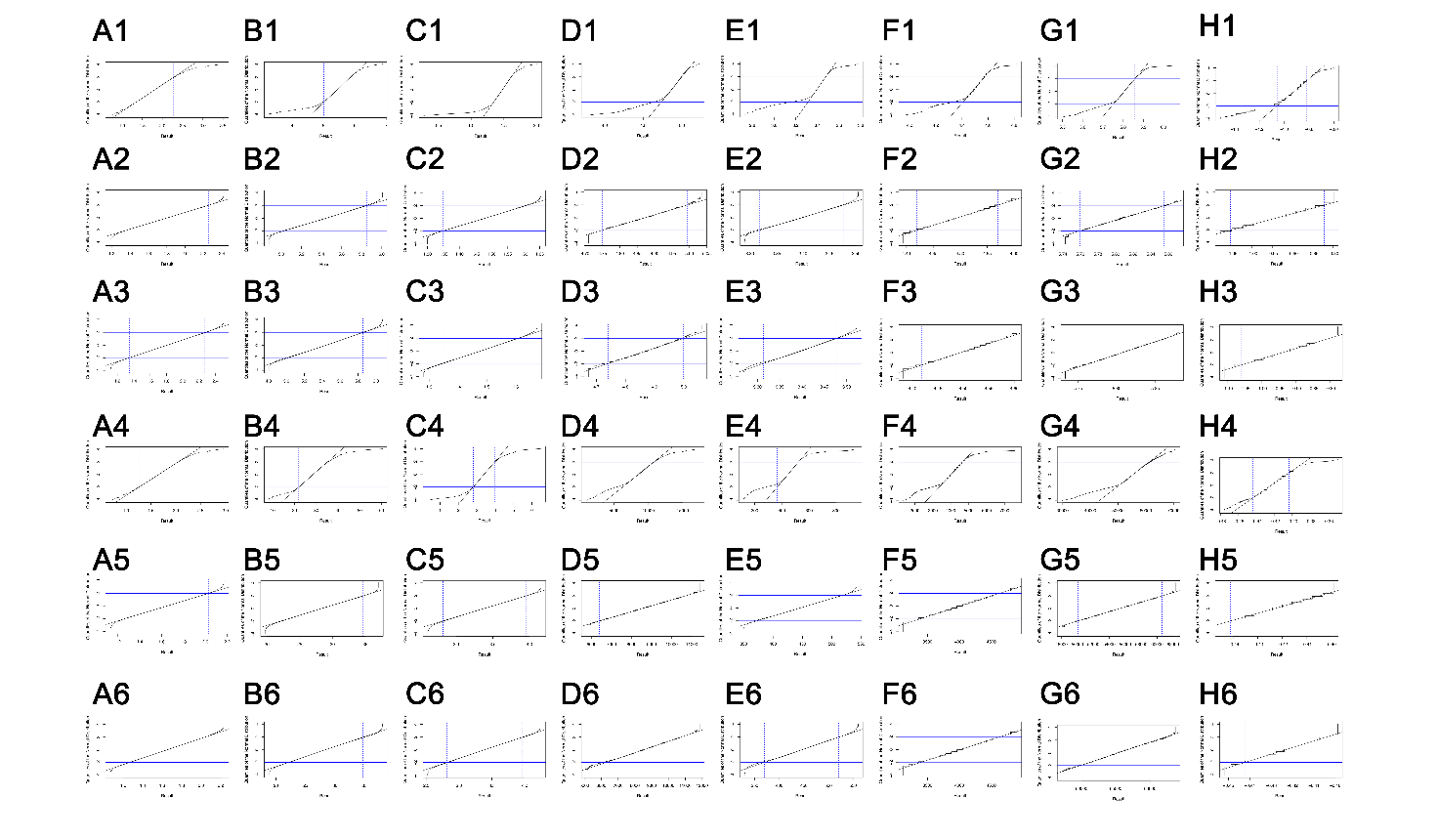
**

**Suppl Fig. 4 Log differences plots with linear fits and data histogram with Bhattacharya fits after different data transformation and outlier removal methods for WBC among males**

A: the log difference plot with the linear fit after log transformation and Dixon method; B: the data histogram with the Bhattacharya fit after log transformation and Dixon method; C: the log difference plot with the linear fit after log transformation and Tukey method; D: the data histogram with the Bhattacharya fit after log transformation and Tukey method; E: the log difference plot with the linear fit after log transformation and mean±3sd method; F: the data histogram with the Bhattacharya fit after log transformation and mean±3sd method; G: the log difference plot with the linear fit after BoxCox transformation and Dixon method; H: the data histogram with the Bhattacharya fit after BoxCox transformation and Dixon method; I: the log difference plot with the linear fit after BoxCox transformation and Tukey method; J: the data histogram with the Bhattacharya fit after BoxCox transformation and Tukey method; K: the log difference plot with the linear fit after BoxCox transformation and mean±3sd method; L: the data histogram with the Bhattacharya fit after BoxCox transformation and mean±3sd method


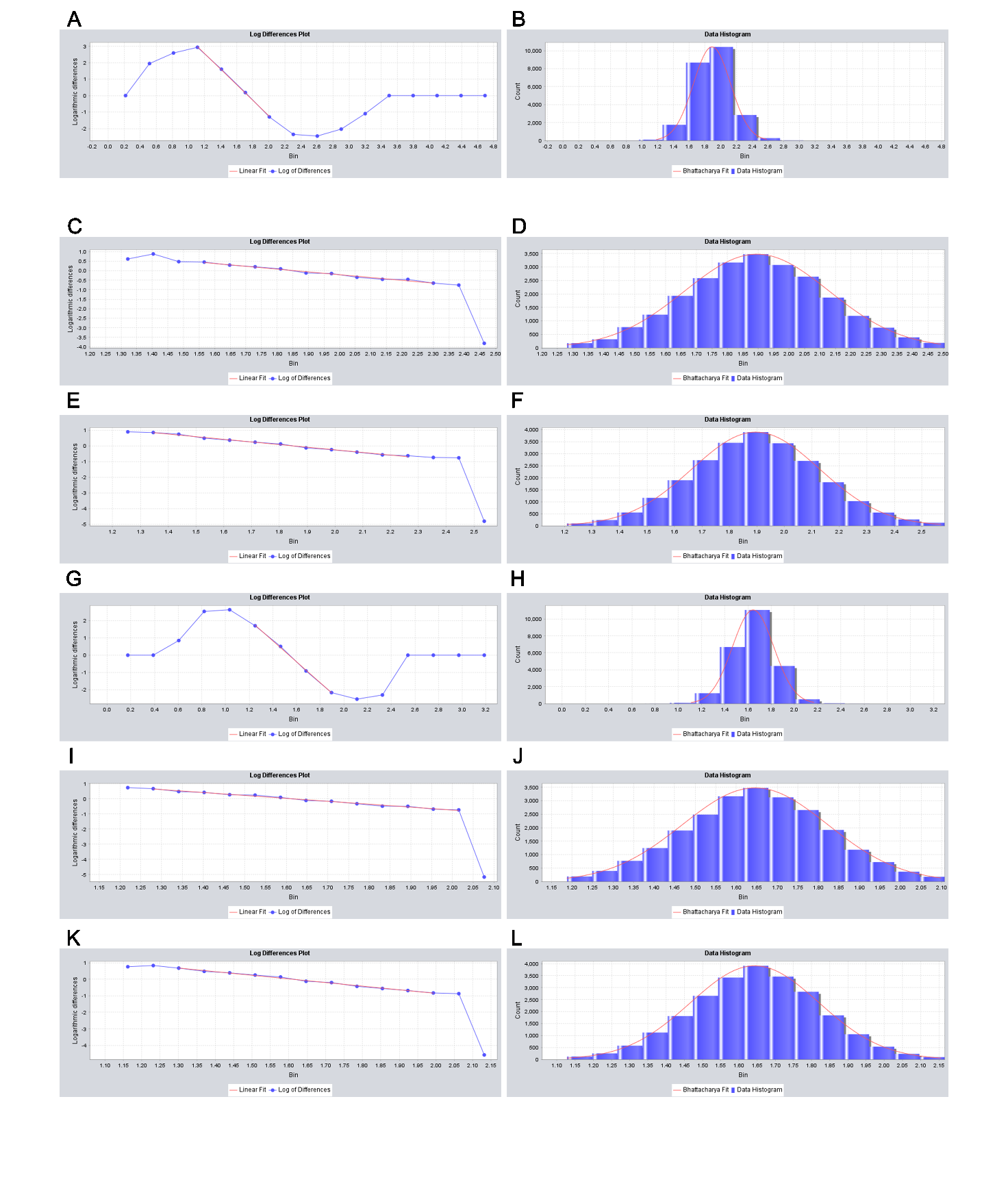


**Suppl Fig. 5 Log differences plots with linear fits and data histogram with Bhattacharya fits after different data transformation and outlier removal methods for PLT among males**

A: the log difference plot with the linear fit after log transformation and Dixon method; B: the data histogram with the Bhattacharya fit after log transformation and Dixon method; C: the log difference plot with the linear fit after log transformation and Tukey method; D: the data histogram with the Bhattacharya fit after log transformation and Tukey method; E: the log difference plot with the linear fit after log transformation and mean±3sd method; F: the data histogram with the Bhattacharya fit after log transformation and mean±3sd method; G: the log difference plot with the linear fit after BoxCox transformation and Dixon method; H: the data histogram with the Bhattacharya fit after BoxCox transformation and Dixon method; I: the log difference plot with the linear fit after BoxCox transformation and Tukey method; J: the data histogram with the Bhattacharya fit after BoxCox transformation and Tukey method; K: the log difference plot with the linear fit after BoxCox transformation and mean±3sd method; L: the data histogram with the Bhattacharya fit after BoxCox transformation and mean±3sd method


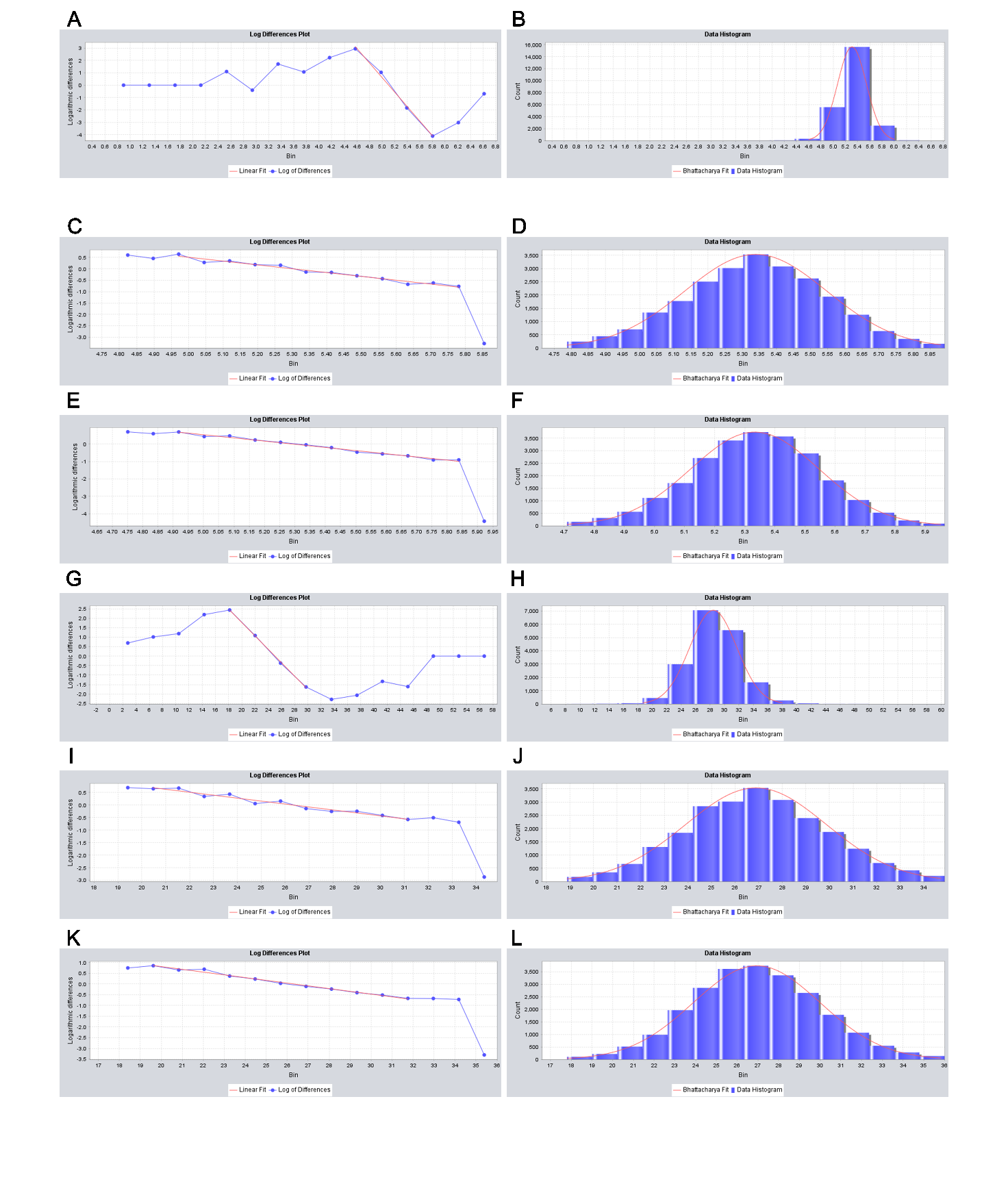


**Suppl Fig. 6 Log differences plots with linear fits and data histogram with Bhattacharya fits after different data transformation and outlier removal methods for RBC among males**

A: the log difference plot with the linear fit after log transformation and Dixon method; B: the data histogram with the Bhattacharya fit after log transformation and Dixon method; C: the log difference plot with the linear fit after log transformation and Tukey method; D: the data histogram with the Bhattacharya fit after log transformation and Tukey method; E: the log difference plot with the linear fit after log transformation and mean±3sd method; F: the data histogram with the Bhattacharya fit after log transformation and mean±3sd method; G: the log difference plot with the linear fit after BoxCox transformation and Dixon method; H: the data histogram with the Bhattacharya fit after BoxCox transformation and Dixon method; I: the log difference plot with the linear fit after BoxCox transformation and Tukey method; J: the data histogram with the Bhattacharya fit after BoxCox transformation and Tukey method; K: the log difference plot with the linear fit after BoxCox transformation and mean±3sd method; L: the data histogram with the Bhattacharya fit after BoxCox transformation and mean±3sd method


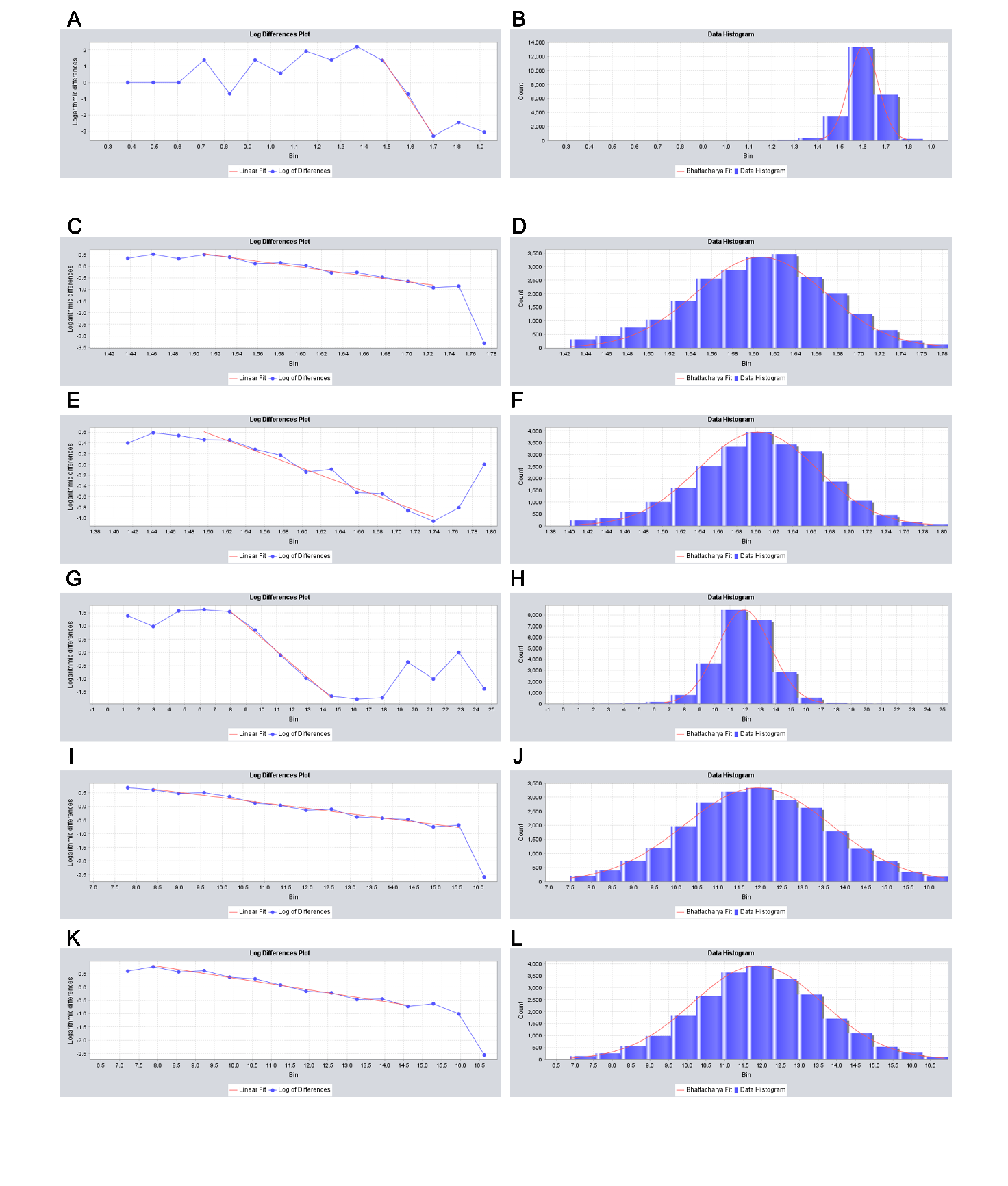


**Suppl Fig. 7 Log differences plots with linear fits and data histogram with Bhattacharya fits after different data transformation and outlier removal methods for HGB among males**

A: the log difference plot with the linear fit after log transformation and Dixon method; B: the data histogram with the Bhattacharya fit after log transformation and Dixon method; C: the log difference plot with the linear fit after log transformation and Tukey method; D: the data histogram with the Bhattacharya fit after log transformation and Tukey method; E: the log difference plot with the linear fit after log transformation and mean±3sd method; F: the data histogram with the Bhattacharya fit after log transformation and mean±3sd method; G: the log difference plot with the linear fit after BoxCox transformation and Dixon method; H: the data histogram with the Bhattacharya fit after BoxCox transformation and Dixon method; I: the log difference plot with the linear fit after BoxCox transformation and Tukey method; J: the data histogram with the Bhattacharya fit after BoxCox transformation and Tukey method; K: the log difference plot with the linear fit after BoxCox transformation and mean±3sd method; L: the data histogram with the Bhattacharya fit after BoxCox transformation and mean±3sd method


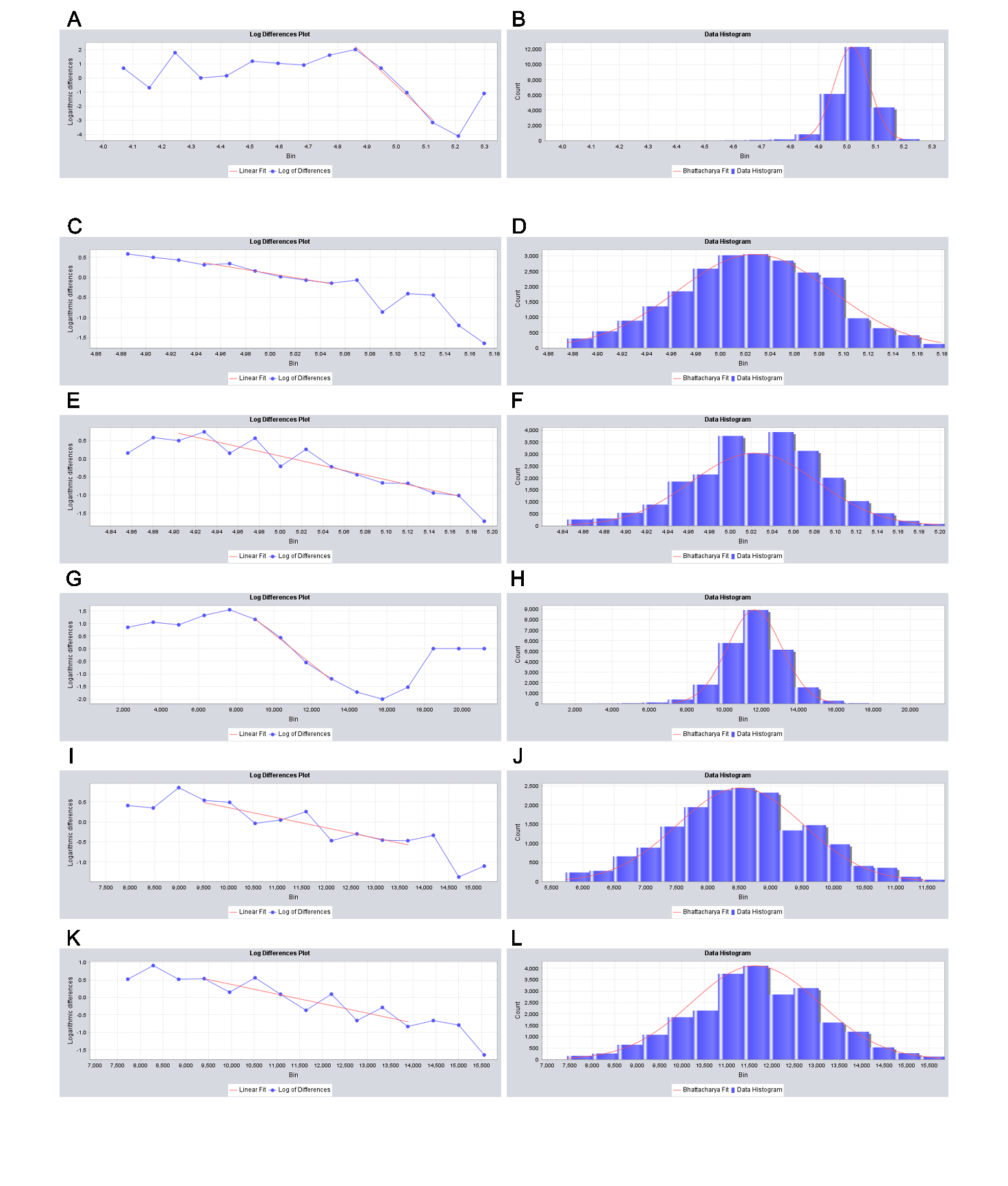


**Suppl Fig. 8 Log differences plots with linear fits and data histogram with Bhattacharya fits after different data transformation and outlier removal methods for MCH among males**

A: the log difference plot with the linear fit after log transformation and Dixon method; B: the data histogram with the Bhattacharya fit after log transformation and Dixon method; C: the log difference plot with the linear fit after log transformation and Tukey method; D: the data histogram with the Bhattacharya fit after log transformation and Tukey method; E: the log difference plot with the linear fit after log transformation and mean±3sd method; F: the data histogram with the Bhattacharya fit after log transformation and mean±3sd method; G: the log difference plot with the linear fit after BoxCox transformation and Dixon method; H: the data histogram with the Bhattacharya fit after BoxCox transformation and Dixon method; I: the log difference plot with the linear fit after BoxCox transformation and Tukey method; J: the data histogram with the Bhattacharya fit after BoxCox transformation and Tukey method; K: the log difference plot with the linear fit after BoxCox transformation and mean±3sd method; L: the data histogram with the Bhattacharya fit after BoxCox transformation and mean±3sd method


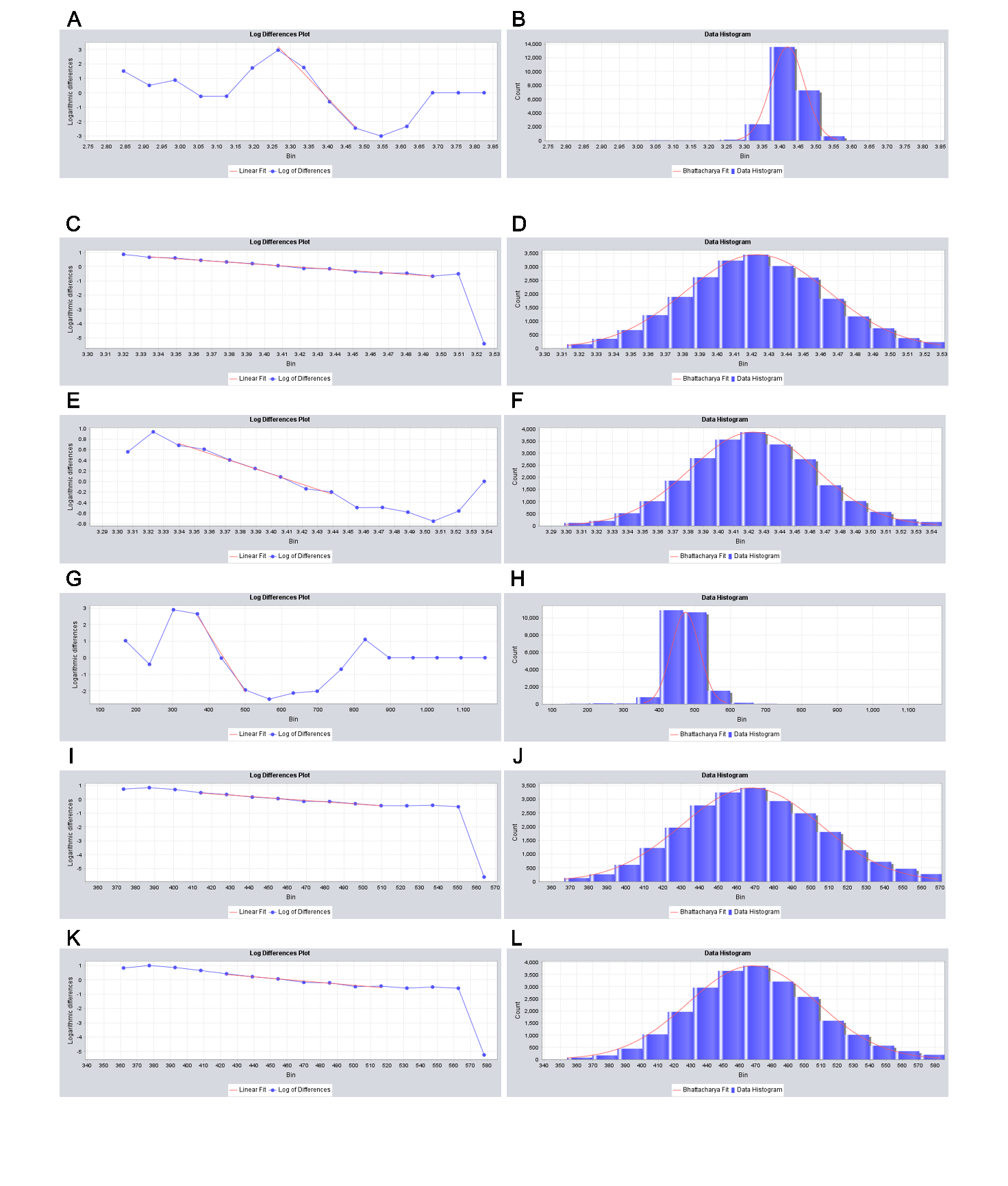


**Suppl Fig. 9 Log differences plots with linear fits and data histogram with Bhattacharya fits after different data transformation and outlier removal methods for MCV among males**

A: the log difference plot with the linear fit after log transformation and Dixon method; B: the data histogram with the Bhattacharya fit after log transformation and Dixon method; C: the log difference plot with the linear fit after log transformation and Tukey method; D: the data histogram with the Bhattacharya fit after log transformation and Tukey method; E: the log difference plot with the linear fit after log transformation and mean±3sd method; F: the data histogram with the Bhattacharya fit after log transformation and mean±3sd method; G: the log difference plot with the linear fit after BoxCox transformation and Dixon method; H: the data histogram with the Bhattacharya fit after BoxCox transformation and Dixon method; I: the log difference plot with the linear fit after BoxCox transformation and Tukey method; J: the data histogram with the Bhattacharya fit after BoxCox transformation and Tukey method; K: the log difference plot with the linear fit after BoxCox transformation and mean±3sd method; L: the data histogram with the Bhattacharya fit after BoxCox transformation and mean±3sd method


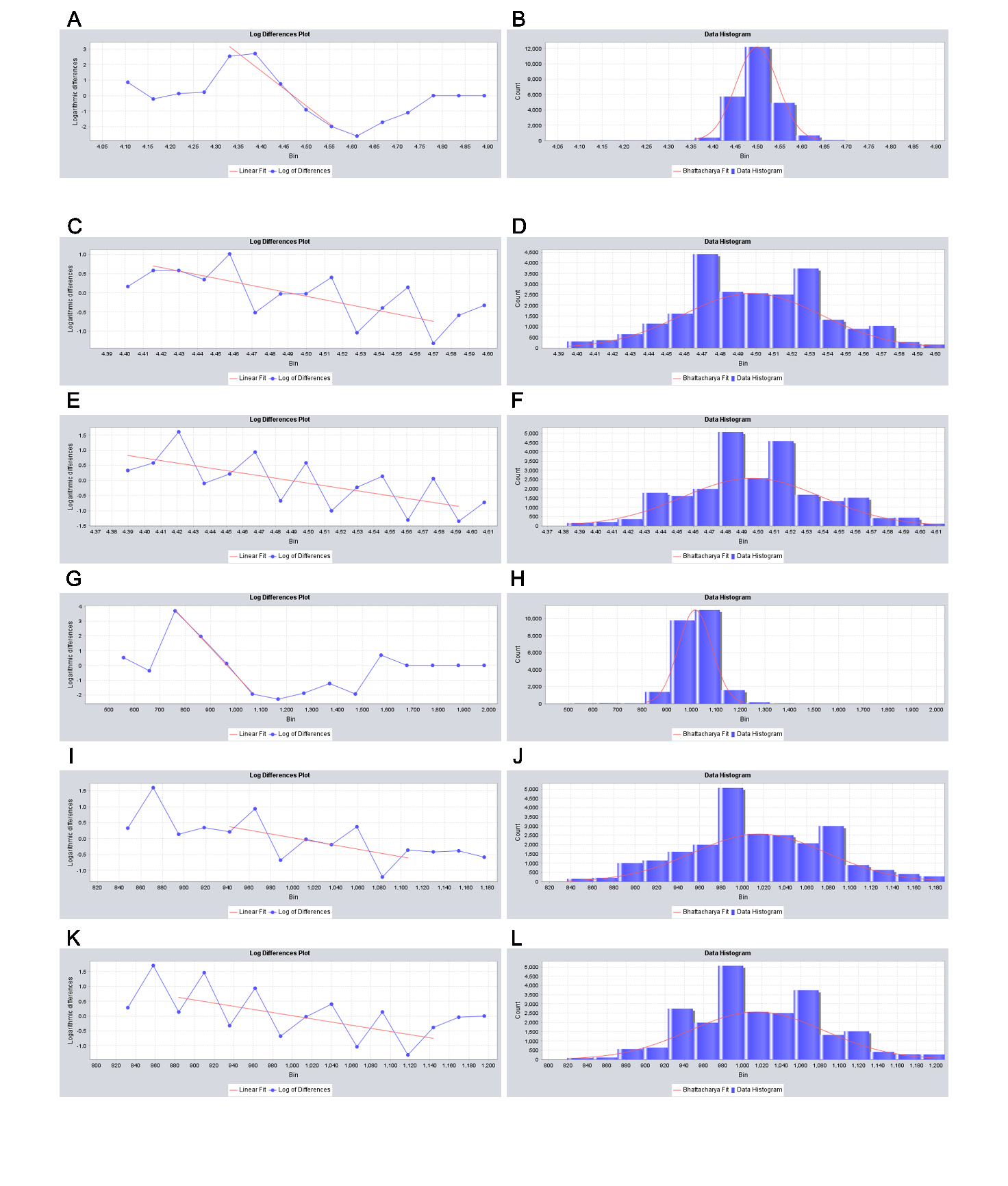


**Suppl Fig. 10 Log differences plots with linear fits and data histogram with Bhattacharya fits after different data transformation and outlier removal methods for MCHC among males**

A: the log difference plot with the linear fit after log transformation and Dixon method; B: the data histogram with the Bhattacharya fit after log transformation and Dixon method; C: the log difference plot with the linear fit after log transformation and Tukey method; D: the data histogram with the Bhattacharya fit after log transformation and Tukey method; E: the log difference plot with the linear fit after log transformation and mean±3sd method; F: the data histogram with the Bhattacharya fit after log transformation and mean±3sd method; G: the log difference plot with the linear fit after BoxCox transformation and Dixon method; H: the data histogram with the Bhattacharya fit after BoxCox transformation and Dixon method; I: the log difference plot with the linear fit after BoxCox transformation and Tukey method; J: the data histogram with the Bhattacharya fit after BoxCox transformation and Tukey method; K: the log difference plot with the linear fit after BoxCox transformation and mean±3sd method; L: the data histogram with the Bhattacharya fit after BoxCox transformation and mean±3sd method


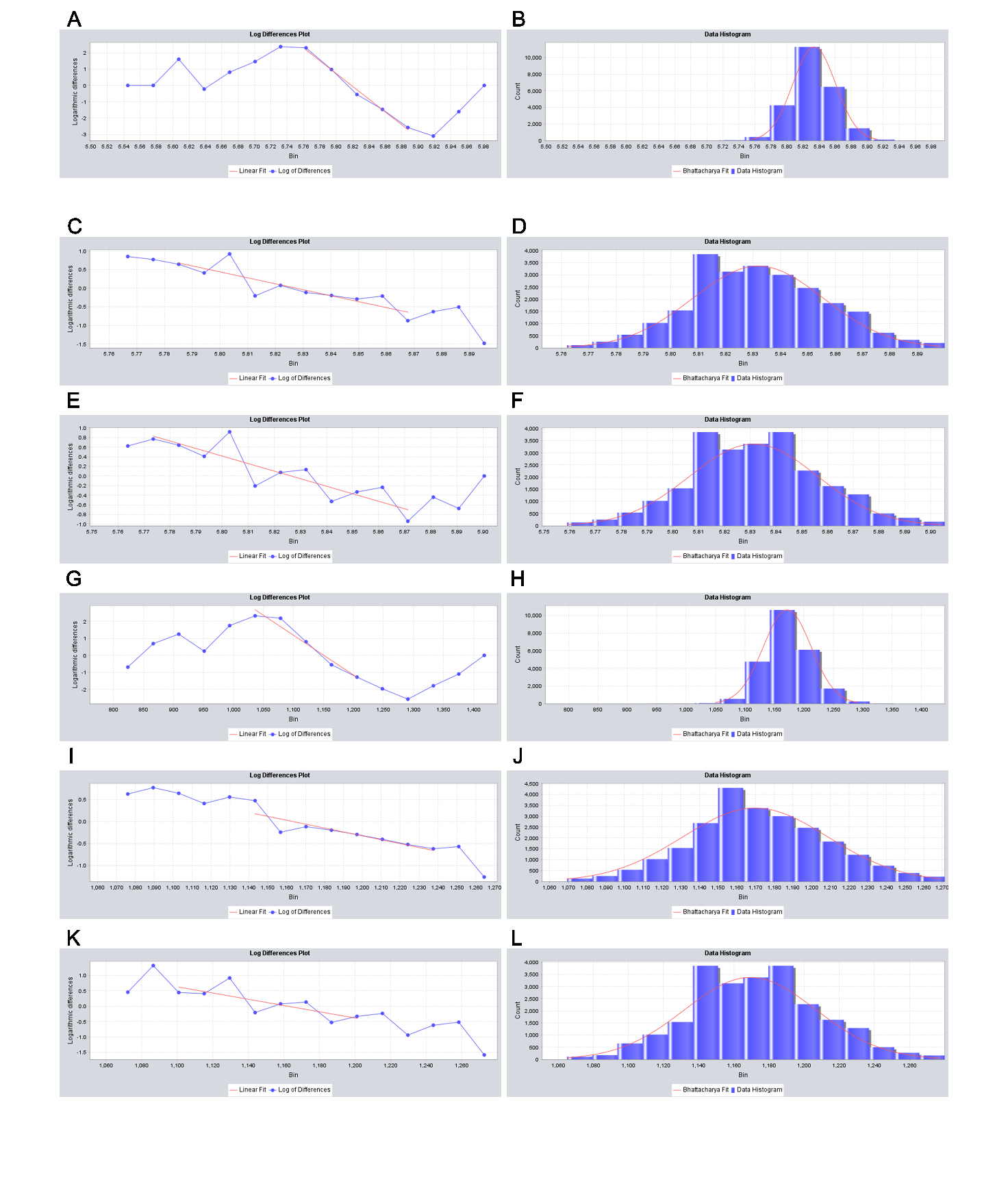


**Suppl Fig. 11 Log differences plots with linear fits and data histogram with Bhattacharya fits after different data transformation and outlier removal methods for HCT among males**

A: the log difference plot with the linear fit after log transformation and Dixon method; B: the data histogram with the Bhattacharya fit after log transformation and Dixon method; C: the log difference plot with the linear fit after log transformation and Tukey method; D: the data histogram with the Bhattacharya fit after log transformation and Tukey method; E: the log difference plot with the linear fit after log transformation and mean±3sd method; F: the data histogram with the Bhattacharya fit after log transformation and mean±3sd method; G: the log difference plot with the linear fit after BoxCox transformation and Dixon method; H: the data histogram with the Bhattacharya fit after BoxCox transformation and Dixon method; I: the log difference plot with the linear fit after BoxCox transformation and Tukey method; J: the data histogram with the Bhattacharya fit after BoxCox transformation and Tukey method; K: the log difference plot with the linear fit after BoxCox transformation and mean±3sd method; L: the data histogram with the Bhattacharya fit after BoxCox transformation and mean±3sd method


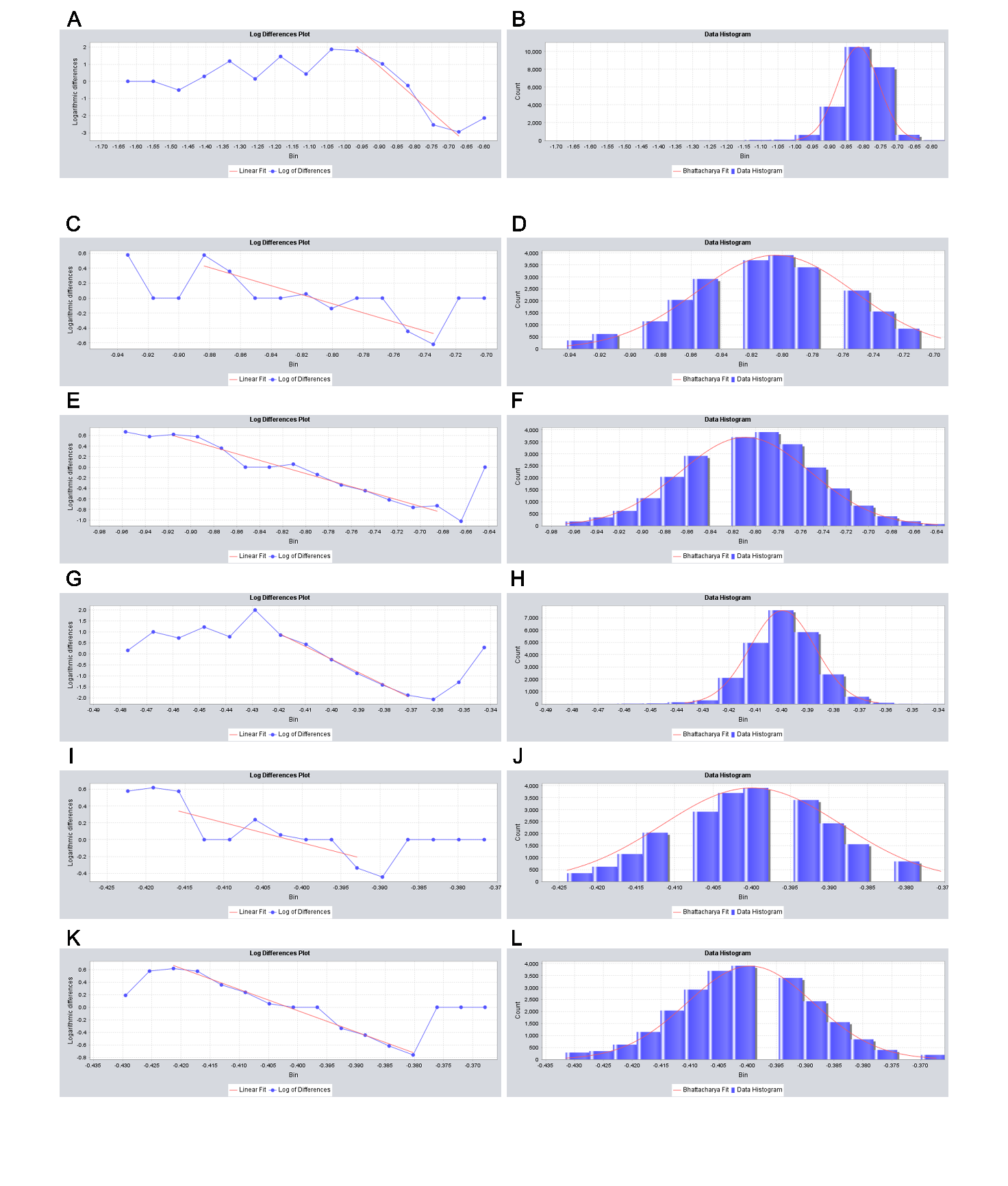


**Suppl Fig. 12 Log differences plots with linear fits and data histogram with Bhattacharya fits after different data transformation and outlier removal methods for WBC among females**

A: the log difference plot with the linear fit after log transformation and Dixon method; B: the data histogram with the Bhattacharya fit after log transformation and Dixon method; C: the log difference plot with the linear fit after log transformation and Tukey method; D: the data histogram with the Bhattacharya fit after log transformation and Tukey method; E: the log difference plot with the linear fit after log transformation and mean±3sd method; F: the data histogram with the Bhattacharya fit after log transformation and mean±3sd method; G: the log difference plot with the linear fit after BoxCox transformation and Dixon method; H: the data histogram with the Bhattacharya fit after BoxCox transformation and Dixon method; I: the log difference plot with the linear fit after BoxCox transformation and Tukey method; J: the data histogram with the Bhattacharya fit after BoxCox transformation and Tukey method; K: the log difference plot with the linear fit after BoxCox transformation and mean±3sd method; L: the data histogram with the Bhattacharya fit after BoxCox transformation and mean±3sd method


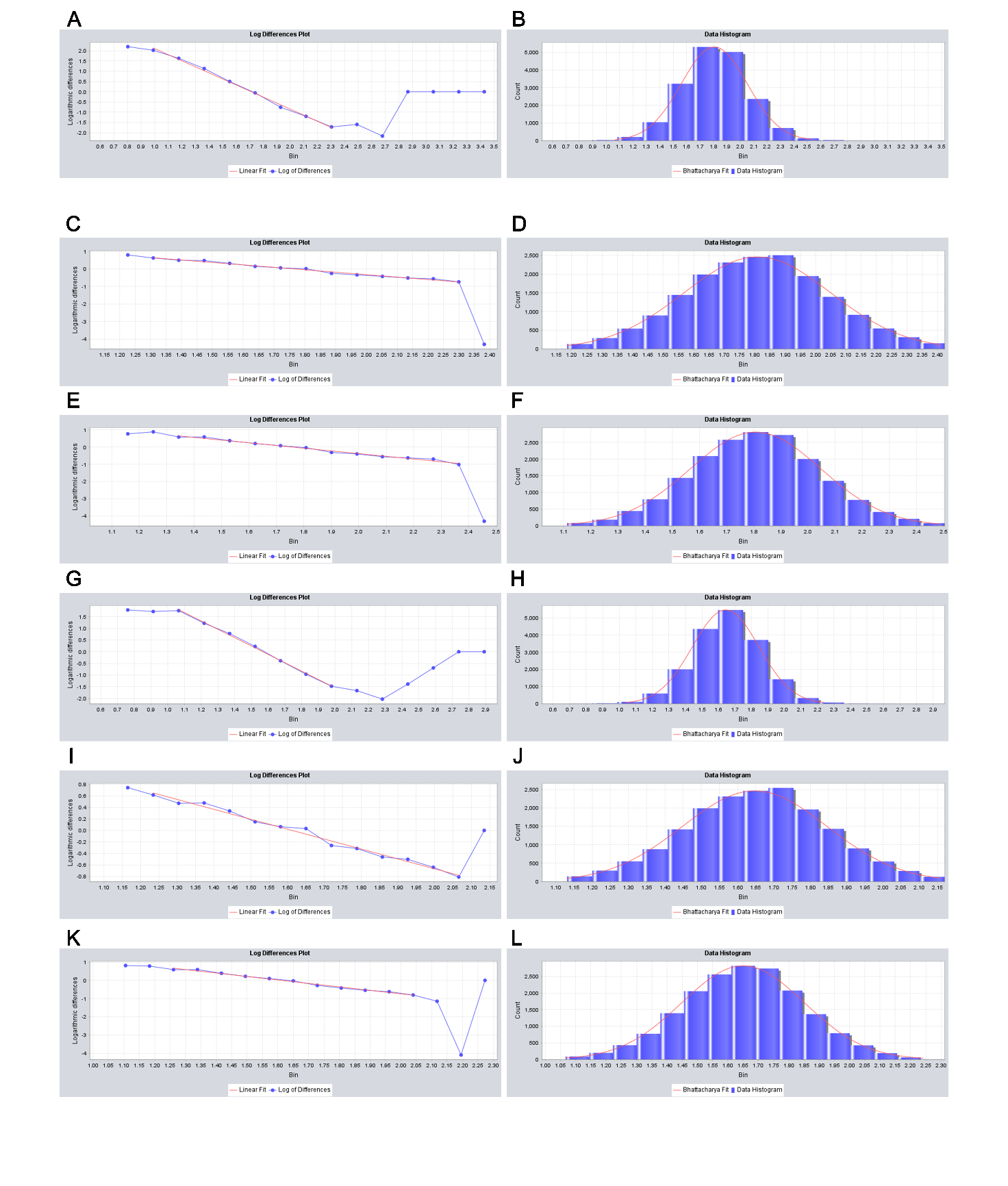


**Suppl Fig. 13 Log differences plots with linear fits and data histogram with Bhattacharya fits after different data transformation and outlier removal methods for PLT among females**

A: the log difference plot with the linear fit after log transformation and Dixon method; B: the data histogram with the Bhattacharya fit after log transformation and Dixon method; C: the log difference plot with the linear fit after log transformation and Tukey method; D: the data histogram with the Bhattacharya fit after log transformation and Tukey method; E: the log difference plot with the linear fit after log transformation and mean±3sd method; F: the data histogram with the Bhattacharya fit after log transformation and mean±3sd method; G: the log difference plot with the linear fit after BoxCox transformation and Dixon method; H: the data histogram with the Bhattacharya fit after BoxCox transformation and Dixon method; I: the log difference plot with the linear fit after BoxCox transformation and Tukey method; J: the data histogram with the Bhattacharya fit after BoxCox transformation and Tukey method; K: the log difference plot with the linear fit after BoxCox transformation and mean±3sd method; L: the data histogram with the Bhattacharya fit after BoxCox transformation and mean±3sd method


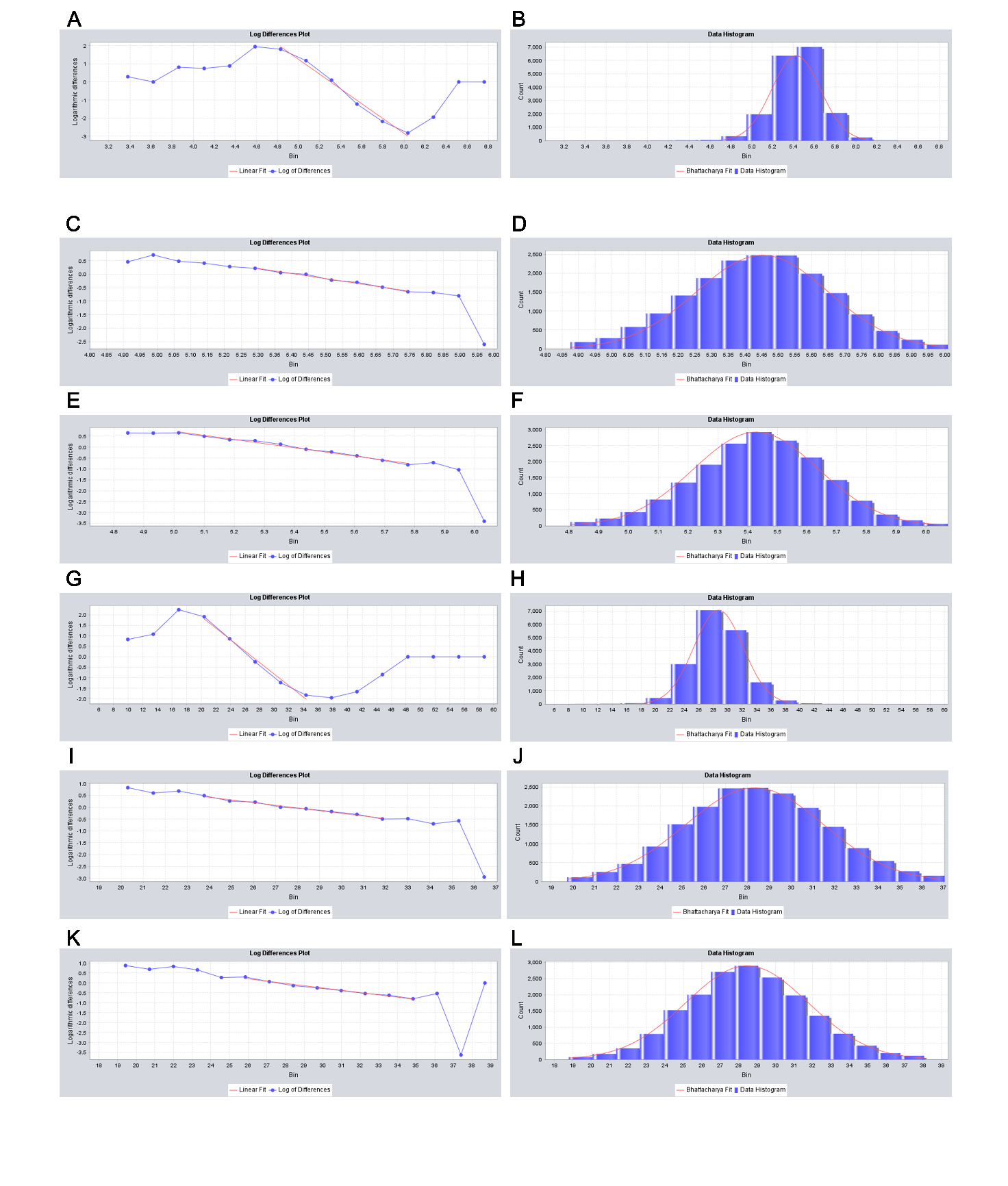


**Suppl Fig. 14 Log differences plots with linear fits and data histogram with Bhattacharya fits after different data transformation and outlier removal methods for RBC among females**

A: the log difference plot with the linear fit after log transformation and Dixon method; B: the data histogram with the Bhattacharya fit after log transformation and Dixon method; C: the log difference plot with the linear fit after log transformation and Tukey method; D: the data histogram with the Bhattacharya fit after log transformation and Tukey method; E: the log difference plot with the linear fit after log transformation and mean±3sd method; F: the data histogram with the Bhattacharya fit after log transformation and mean±3sd method; G: the log difference plot with the linear fit after BoxCox transformation and Dixon method; H: the data histogram with the Bhattacharya fit after BoxCox transformation and Dixon method; I: the log difference plot with the linear fit after BoxCox transformation and Tukey method; J: the data histogram with the Bhattacharya fit after BoxCox transformation and Tukey method; K: the log difference plot with the linear fit after BoxCox transformation and mean±3sd method; L: the data histogram with the Bhattacharya fit after BoxCox transformation and mean±3sd method


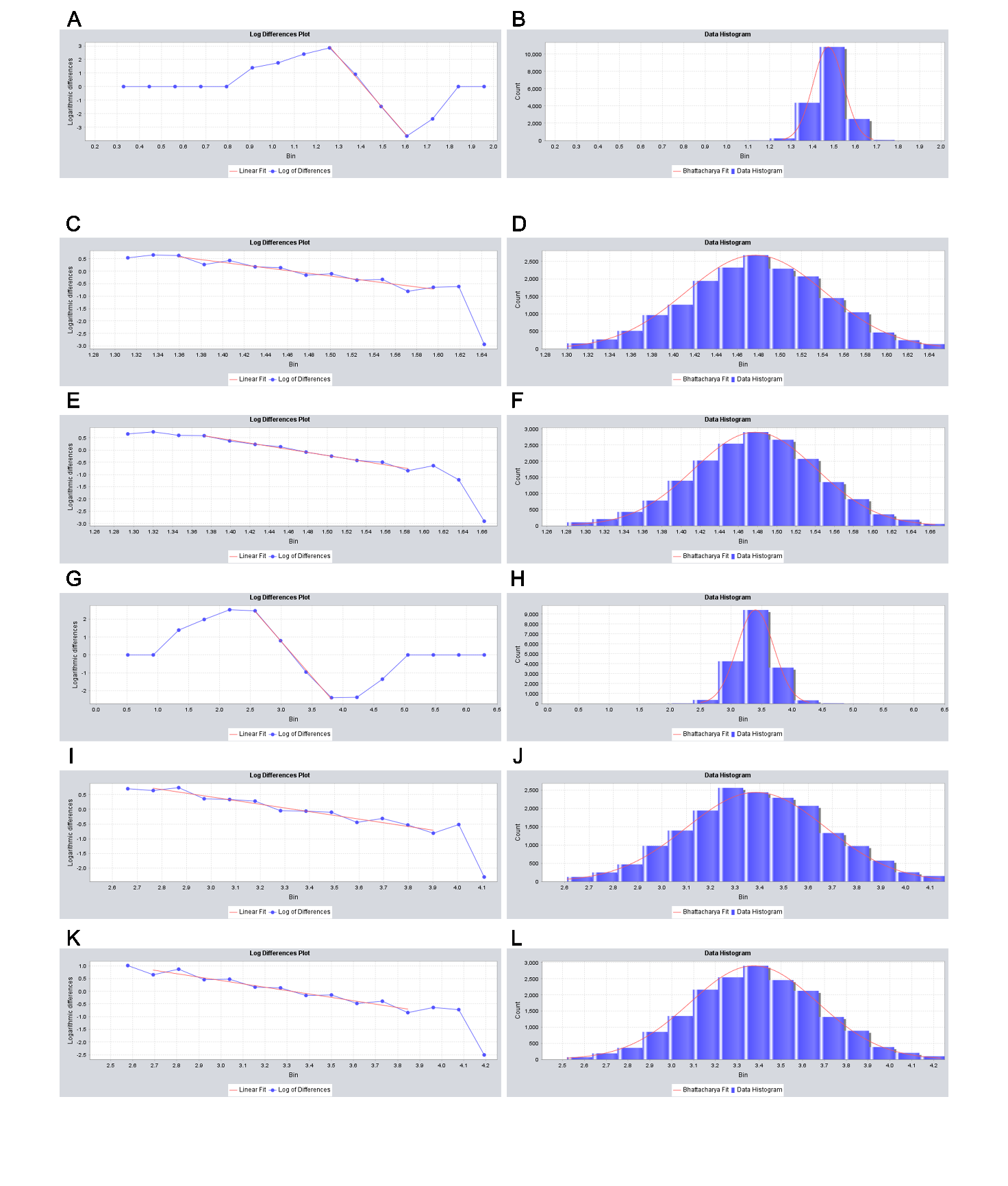


**Suppl Fig. 15 Log differences plots with linear fits and data histogram with Bhattacharya fits after different data transformation and outlier removal methods for HGB among females**

A: the log difference plot with the linear fit after log transformation and Dixon method; B: the data histogram with the Bhattacharya fit after log transformation and Dixon method; C: the log difference plot with the linear fit after log transformation and Tukey method; D: the data histogram with the Bhattacharya fit after log transformation and Tukey method; E: the log difference plot with the linear fit after log transformation and mean±3sd method; F: the data histogram with the Bhattacharya fit after log transformation and mean±3sd method; G: the log difference plot with the linear fit after BoxCox transformation and Dixon method; H: the data histogram with the Bhattacharya fit after BoxCox transformation and Dixon method; I: the log difference plot with the linear fit after BoxCox transformation and Tukey method; J: the data histogram with the Bhattacharya fit after BoxCox transformation and Tukey method; K: the log difference plot with the linear fit after BoxCox transformation and mean±3sd method; L: the data histogram with the Bhattacharya fit after BoxCox transformation and mean±3sd method


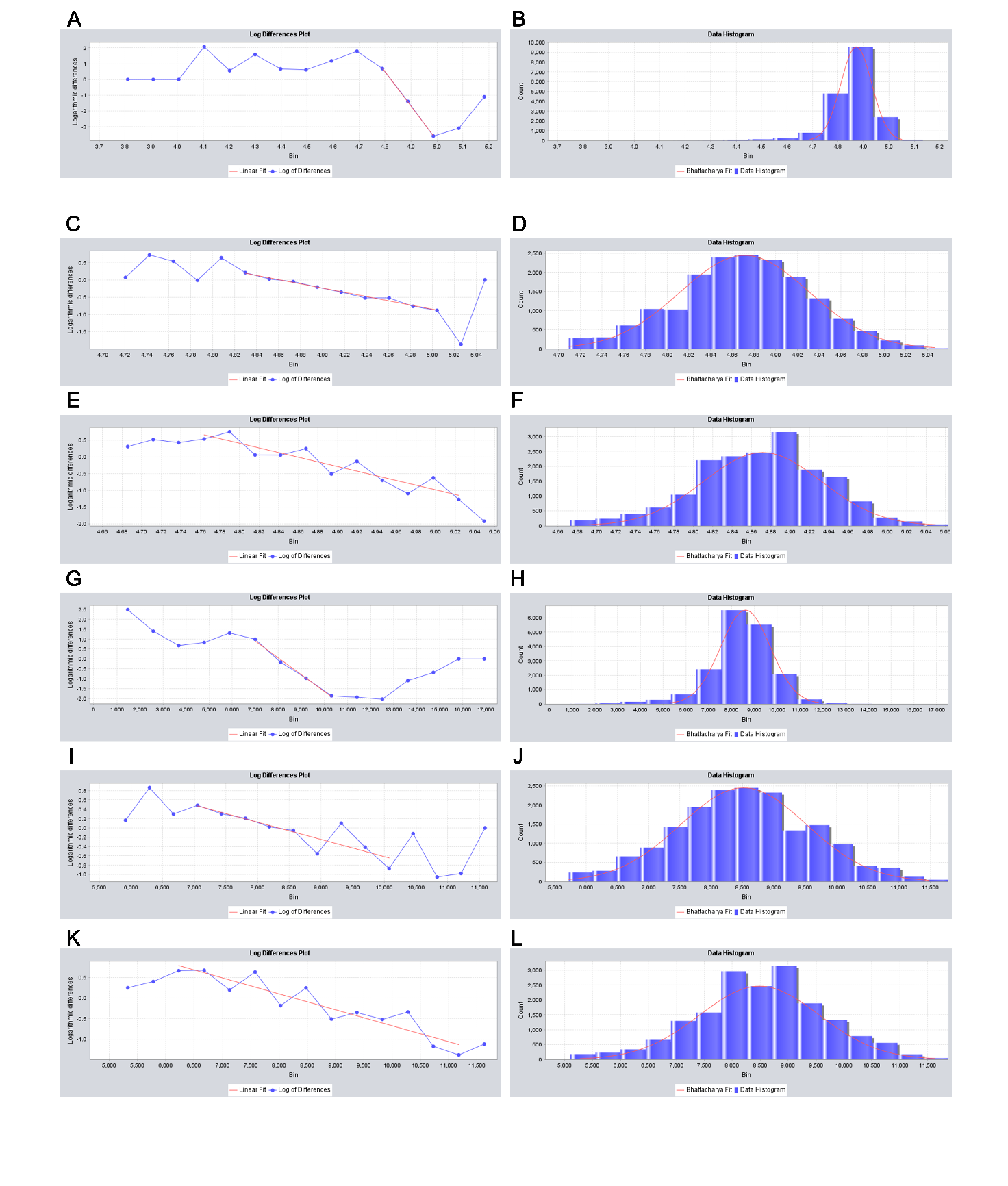


**Suppl Fig. 16 Log differences plots with linear fits and data histogram with Bhattacharya fits after different data transformation and outlier removal methods for MCH among females**

A: the log difference plot with the linear fit after log transformation and Dixon method; B: the data histogram with the Bhattacharya fit after log transformation and Dixon method; C: the log difference plot with the linear fit after log transformation and Tukey method; D: the data histogram with the Bhattacharya fit after log transformation and Tukey method; E: the log difference plot with the linear fit after log transformation and mean±3sd method; F: the data histogram with the Bhattacharya fit after log transformation and mean±3sd method; G: the log difference plot with the linear fit after BoxCox transformation and Dixon method; H: the data histogram with the Bhattacharya fit after BoxCox transformation and Dixon method; I: the log difference plot with the linear fit after BoxCox transformation and Tukey method; J: the data histogram with the Bhattacharya fit after BoxCox transformation and Tukey method; K: the log difference plot with the linear fit after BoxCox transformation and mean±3sd method; L: the data histogram with the Bhattacharya fit after BoxCox transformation and mean±3sd method


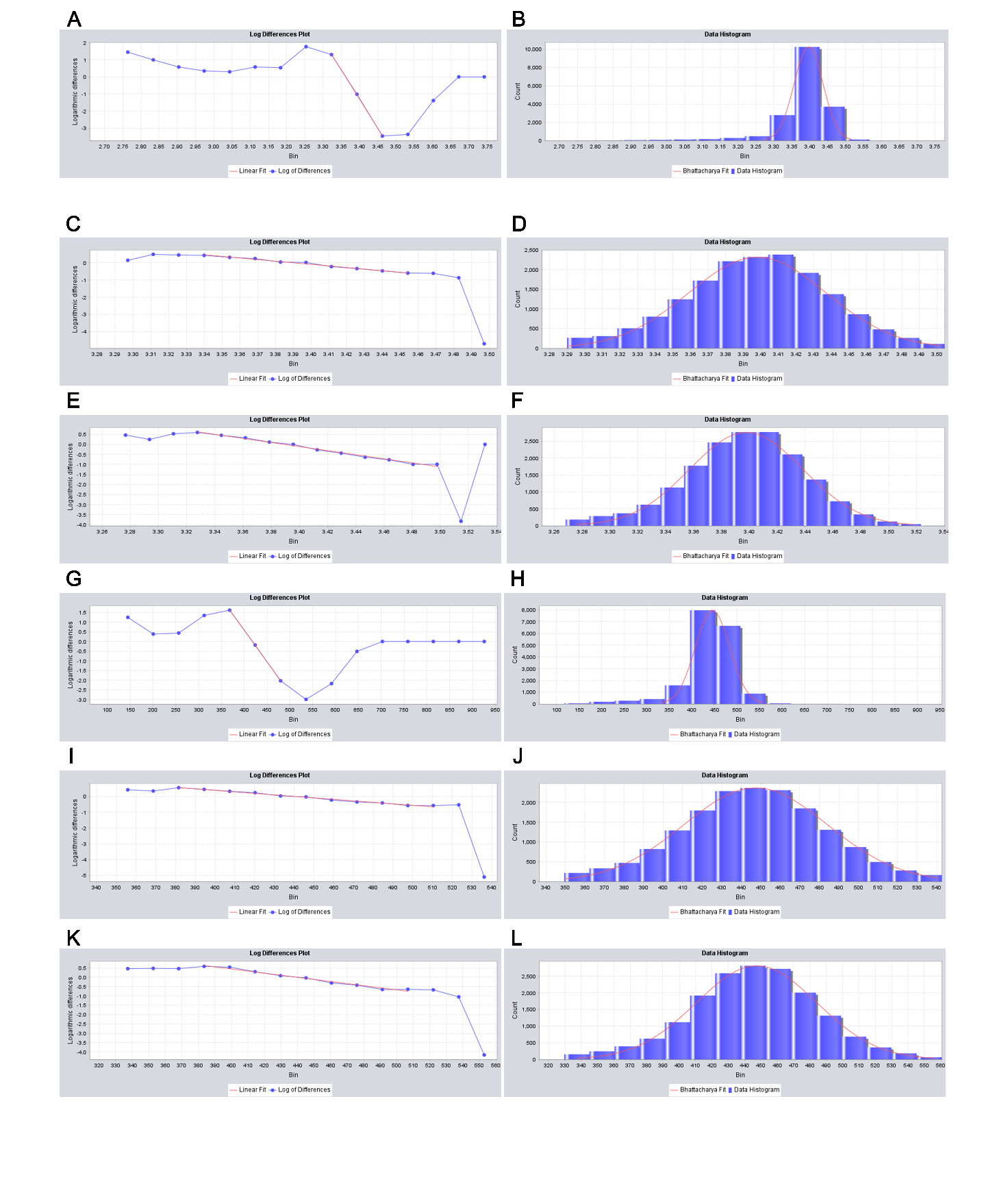


**Suppl Fig. 17 Log differences plots with linear fits and data histogram with Bhattacharya fits after different data transformation and outlier removal methods for MCV among females**

A: the log difference plot with the linear fit after log transformation and Dixon method; B: the data histogram with the Bhattacharya fit after log transformation and Dixon method; C: the log difference plot with the linear fit after log transformation and Tukey method; D: the data histogram with the Bhattacharya fit after log transformation and Tukey method; E: the log difference plot with the linear fit after log transformation and mean±3sd method; F: the data histogram with the Bhattacharya fit after log transformation and mean±3sd method; G: the log difference plot with the linear fit after BoxCox transformation and Dixon method; H: the data histogram with the Bhattacharya fit after BoxCox transformation and Dixon method; I: the log difference plot with the linear fit after BoxCox transformation and Tukey method; J: the data histogram with the Bhattacharya fit after BoxCox transformation and Tukey method; K: the log difference plot with the linear fit after BoxCox transformation and mean±3sd method; L: the data histogram with the Bhattacharya fit after BoxCox transformation and mean±3sd method


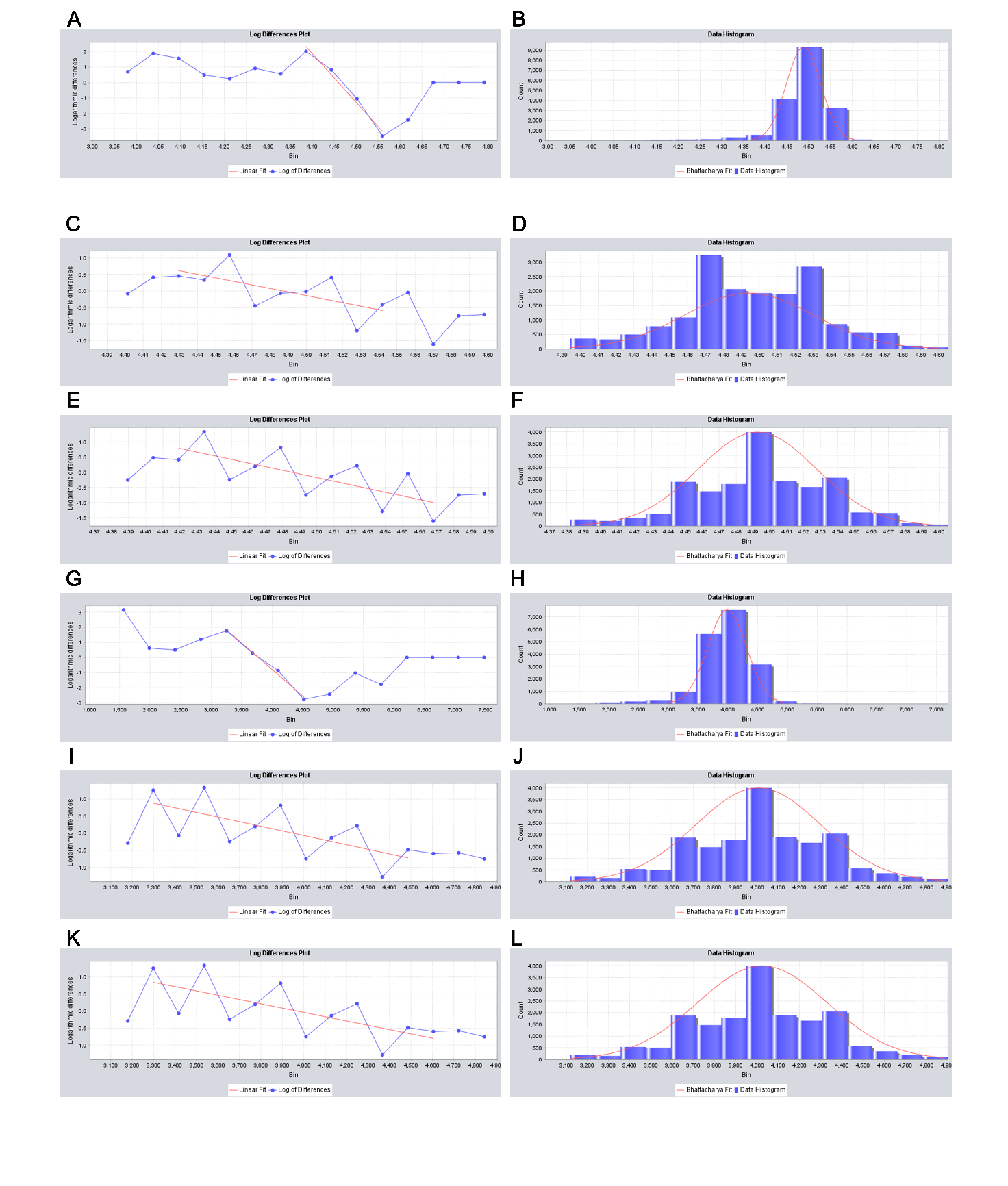


**Suppl Fig. 18 Log differences plots with linear fits and data histogram with Bhattacharya fits after different data transformation and outlier removal methods for MCHC among females**

A: the log difference plot with the linear fit after log transformation and Dixon method; B: the data histogram with the Bhattacharya fit after log transformation and Dixon method; C: the log difference plot with the linear fit after log transformation and Tukey method; D: the data histogram with the Bhattacharya fit after log transformation and Tukey method; E: the log difference plot with the linear fit after log transformation and mean±3sd method; F: the data histogram with the Bhattacharya fit after log transformation and mean±3sd method; G: the log difference plot with the linear fit after BoxCox transformation and Dixon method; H: the data histogram with the Bhattacharya fit after BoxCox transformation and Dixon method; I: the log difference plot with the linear fit after BoxCox transformation and Tukey method; J: the data histogram with the Bhattacharya fit after BoxCox transformation and Tukey method; K: the log difference plot with the linear fit after BoxCox transformation and mean±3sd method; L: the data histogram with the Bhattacharya fit after BoxCox transformation and mean±3sd method


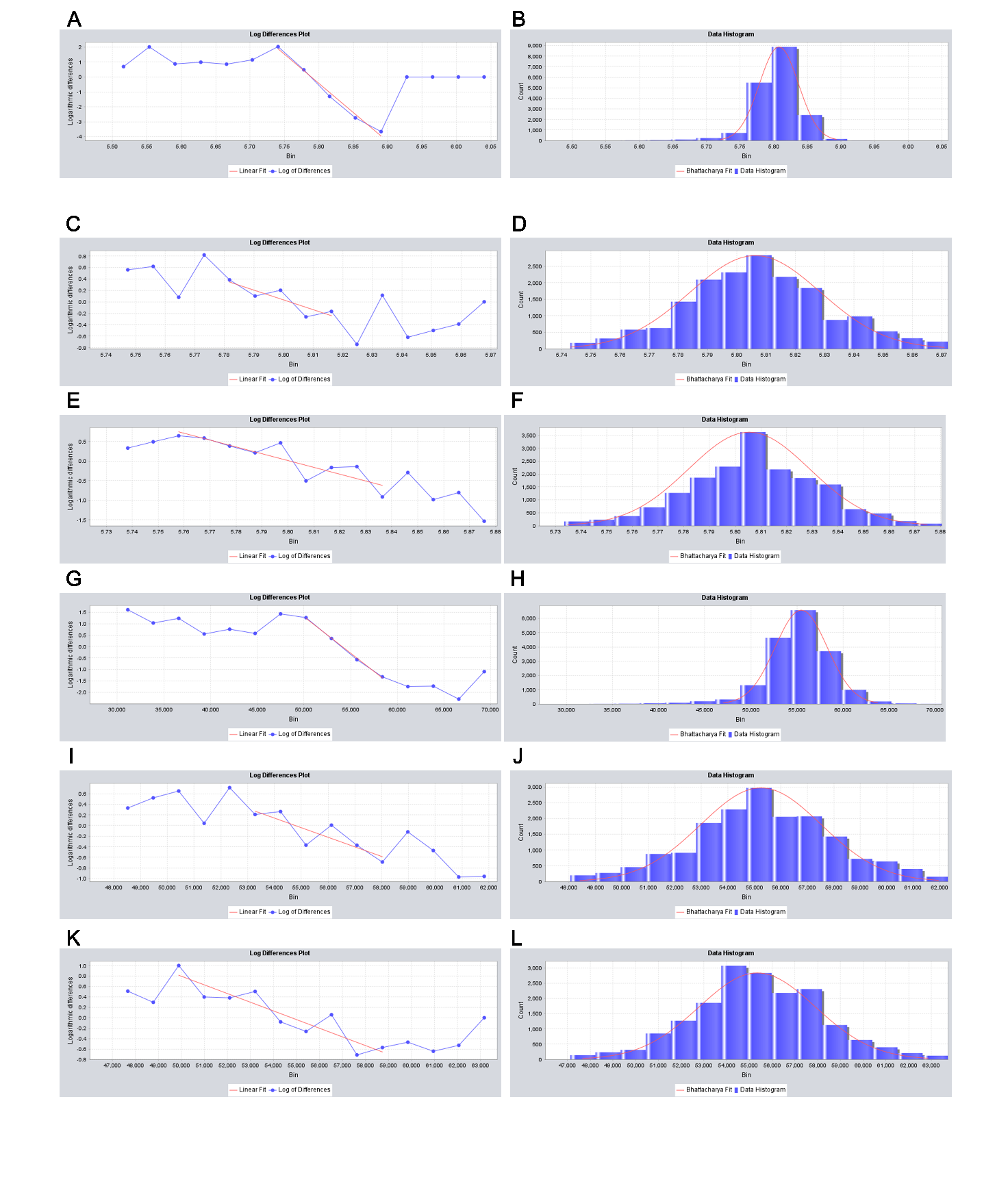


**Suppl Fig. 19 Log differences plots with linear fits and data histogram with Bhattacharya fits after different data transformation and outlier removal methods for HCT among females**

A: the log difference plot with the linear fit after log transformation and Dixon method; B: the data histogram with the Bhattacharya fit after log transformation and Dixon method; C: the log difference plot with the linear fit after log transformation and Tukey method; D: the data histogram with the Bhattacharya fit after log transformation and Tukey method; E: the log difference plot with the linear fit after log transformation and mean±3sd method; F: the data histogram with the Bhattacharya fit after log transformation and mean±3sd method; G: the log difference plot with the linear fit after BoxCox transformation and Dixon method; H: the data histogram with the Bhattacharya fit after BoxCox transformation and Dixon method; I: the log difference plot with the linear fit after BoxCox transformation and Tukey method; J: the data histogram with the Bhattacharya fit after BoxCox transformation and Tukey method; K: the log difference plot with the linear fit after BoxCox transformation and mean±3sd method; L: the data histogram with the Bhattacharya fit after BoxCox transformation and mean±3sd method


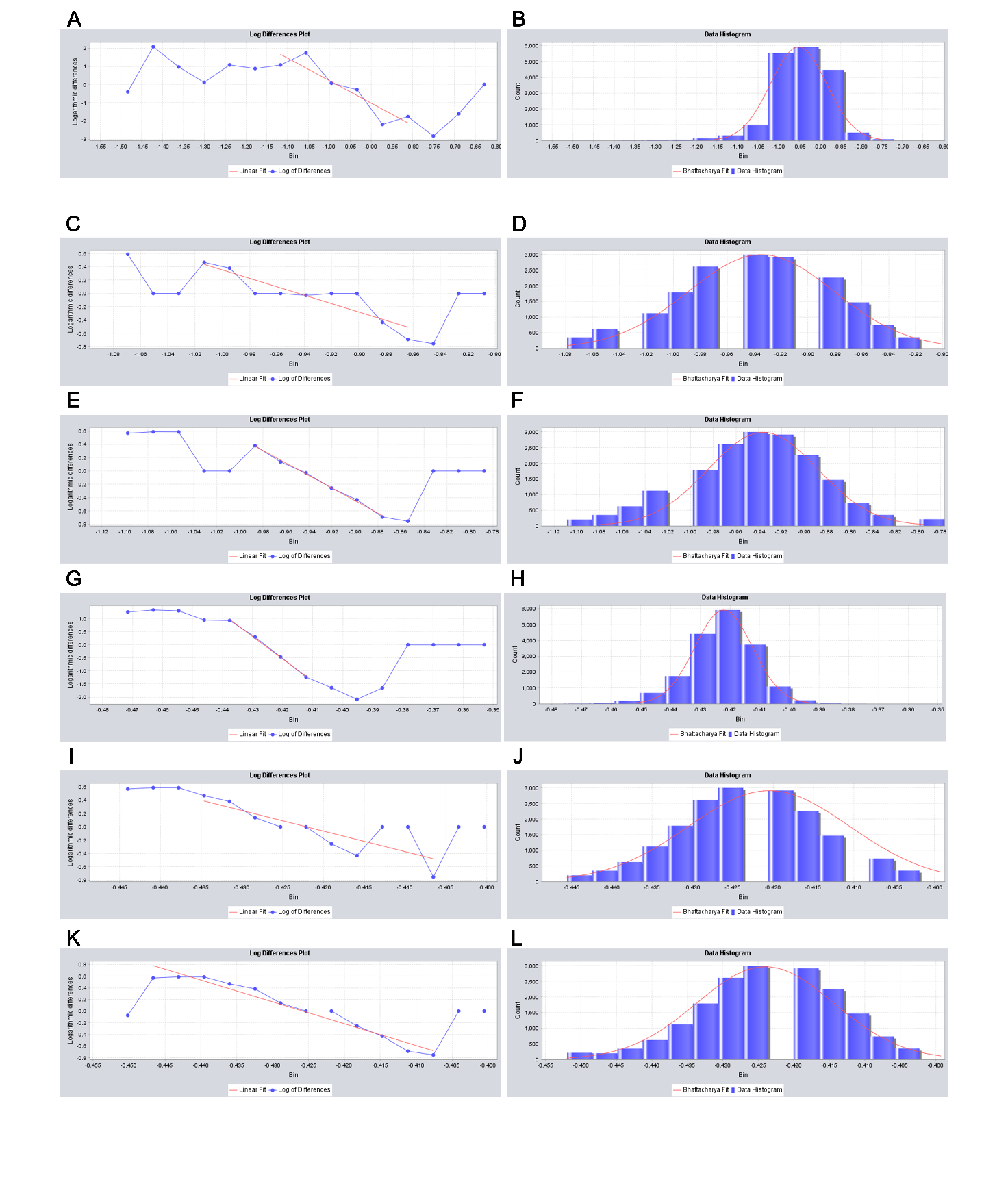


**Suppl Fig. 20 Estimated distributions of reference intervals in a “contaminated” dataset for CBC parameters after different combinations of data transformation and outlier removal methods among males**

A-H represent A: WBC (×10^9^/L); B: PLT (×10^9^/L); C: RBC (×10^12^/L); D: HGB (g/L); E: MCH (pg); F: MCV (fL); G: MCHC (g/L); H: HCT (L/L), respectively. 1-6 represent 1: log+Dixon; 2: log+Tukey; 3: log+mean±3sd; 4: BoxCox+Dixon; 5: BoxCox+Tukey; 6: BoxCox+mean±3sd, respectively.

**
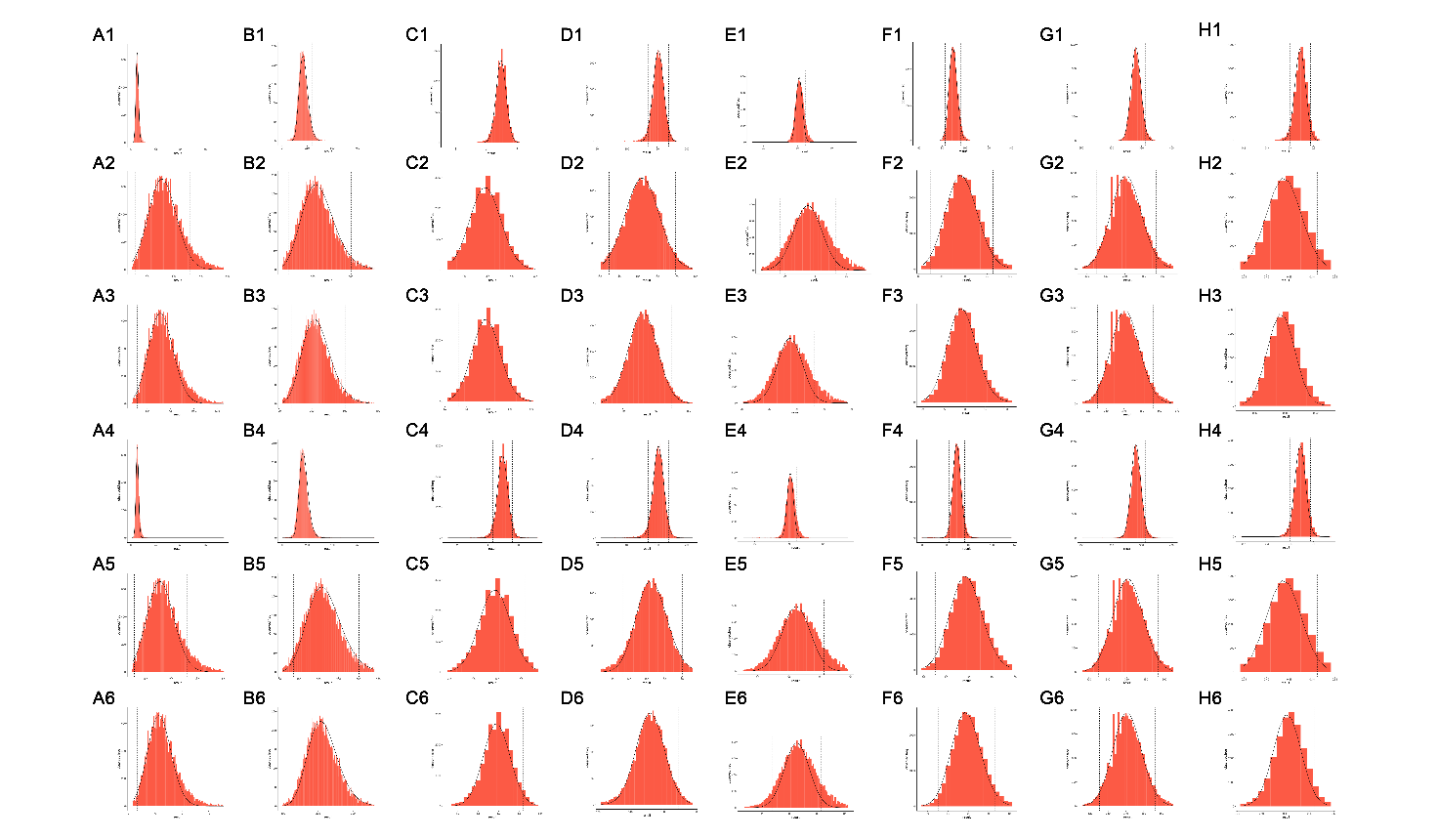
**

**Suppl Fig. 21 Estimated distributions of reference intervals in a “contaminated” dataset for CBC parameters after different combinations of data transformation and outlier removal methods among females**

A-H represent A: WBC (×10^9^/L); B: PLT (×10^9^/L); C: RBC (×10^12^/L); D: HGB (g/L); E: MCH (pg); F: MCV (fL); G: MCHC (g/L); H: HCT (L/L), respectively. 1-6 represent 1: log+Dixon; 2: log+Tukey; 3: log+mean±3sd; 4: BoxCox+Dixon; 5: BoxCox+Tukey; 6: BoxCox+mean±3sd, respectively.

**
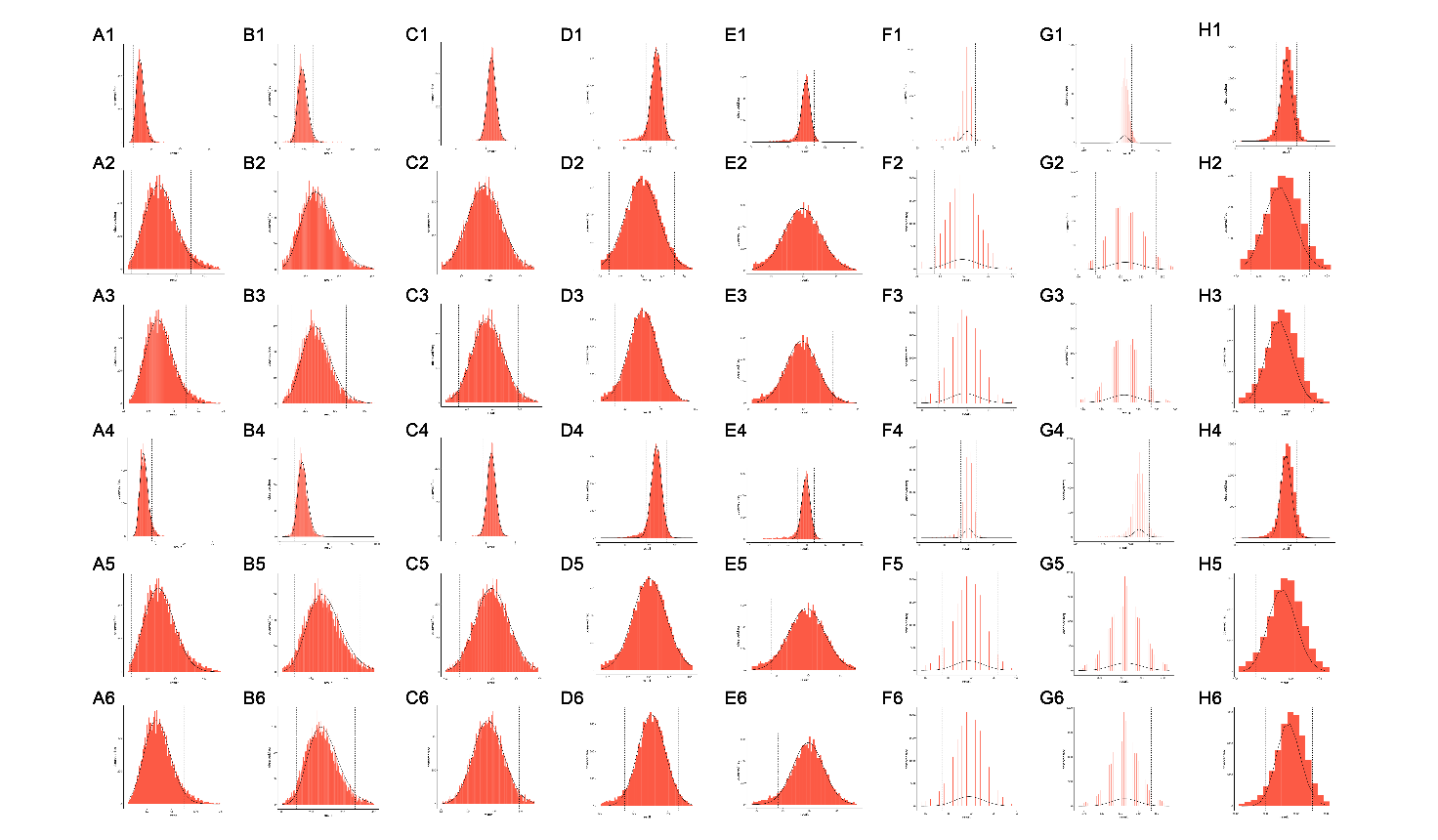
**

**Suppl Fig. 22 Comparison of RIs (WBC, PLT, RBC, HGB) for females by 30 indirect methods with calculation of bias at reference limits**

A: WBC (×10^9^/L); B: PLT (×10^9^/L); C: RBC (×10^12^/L); D: HGB (g/L). LL = lower reference limit. UL = upper reference limit. d% between LL = relative deviation of lower reference limit between indirect and direct methods. d% between UL = relative deviation of upper reference limit between indirect and direct methods. BR for LL = bias ratio at LL. BR for UL = bias ratio at UL.


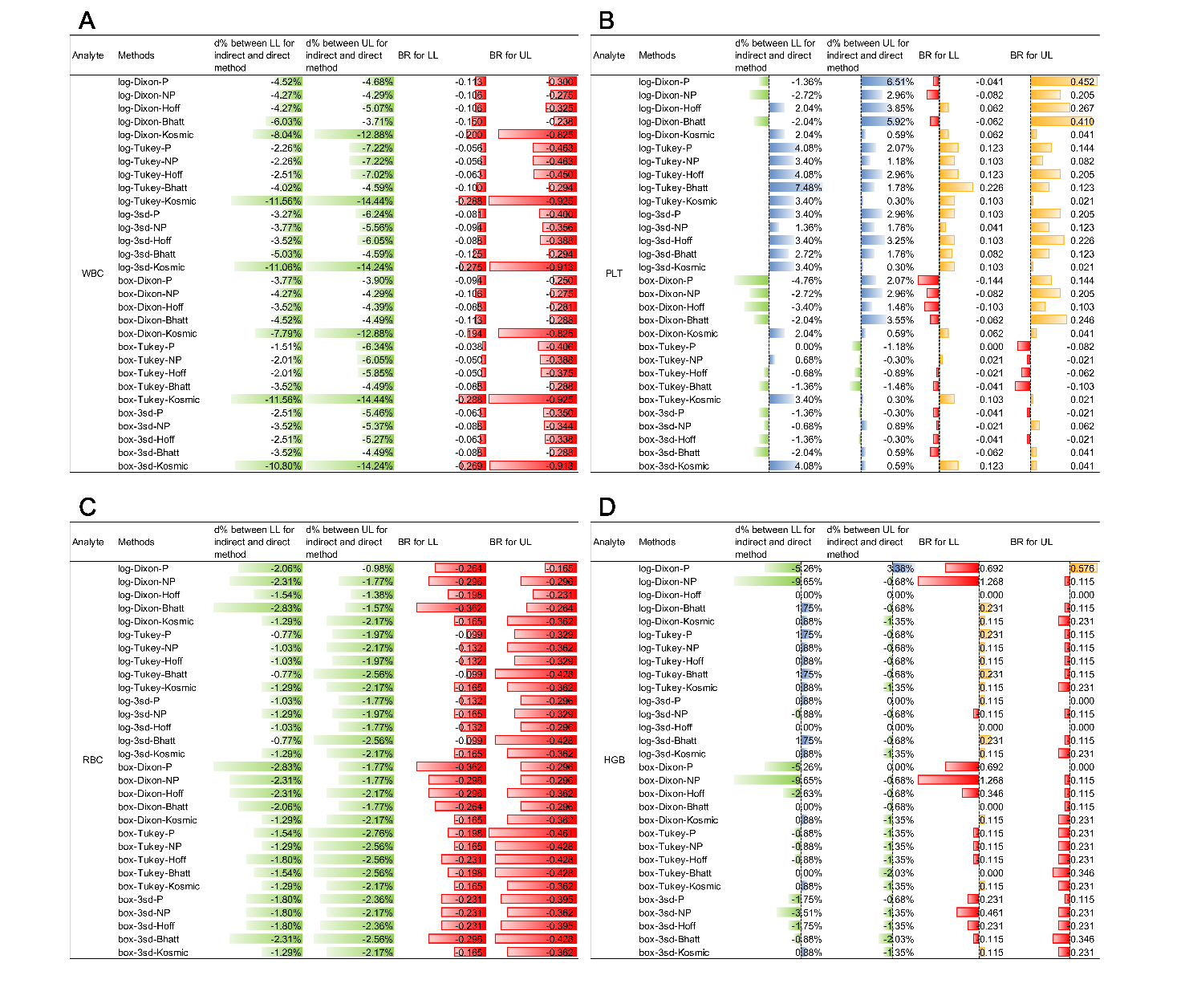


**Suppl Fig. 23 Comparison of RIs (MCH, MCV, MCHC, HCT) for females by 30 indirect methods with calculation of bias at reference limits**

A: MCH (pg); B: MCV (fL); C: MCHC (g/L); D: HCT (L/L). LL = lower reference limit. UL = upper reference limit. d% between LL for indirect and direct method = relative deviation of lower reference limit between indirect and direct methods. d% between UL for indirect and direct method = relative deviation of upper reference limit between indirect and direct methods. BR for LL = bias ratio at LL. BR for UL = bias ratio at UL.

**
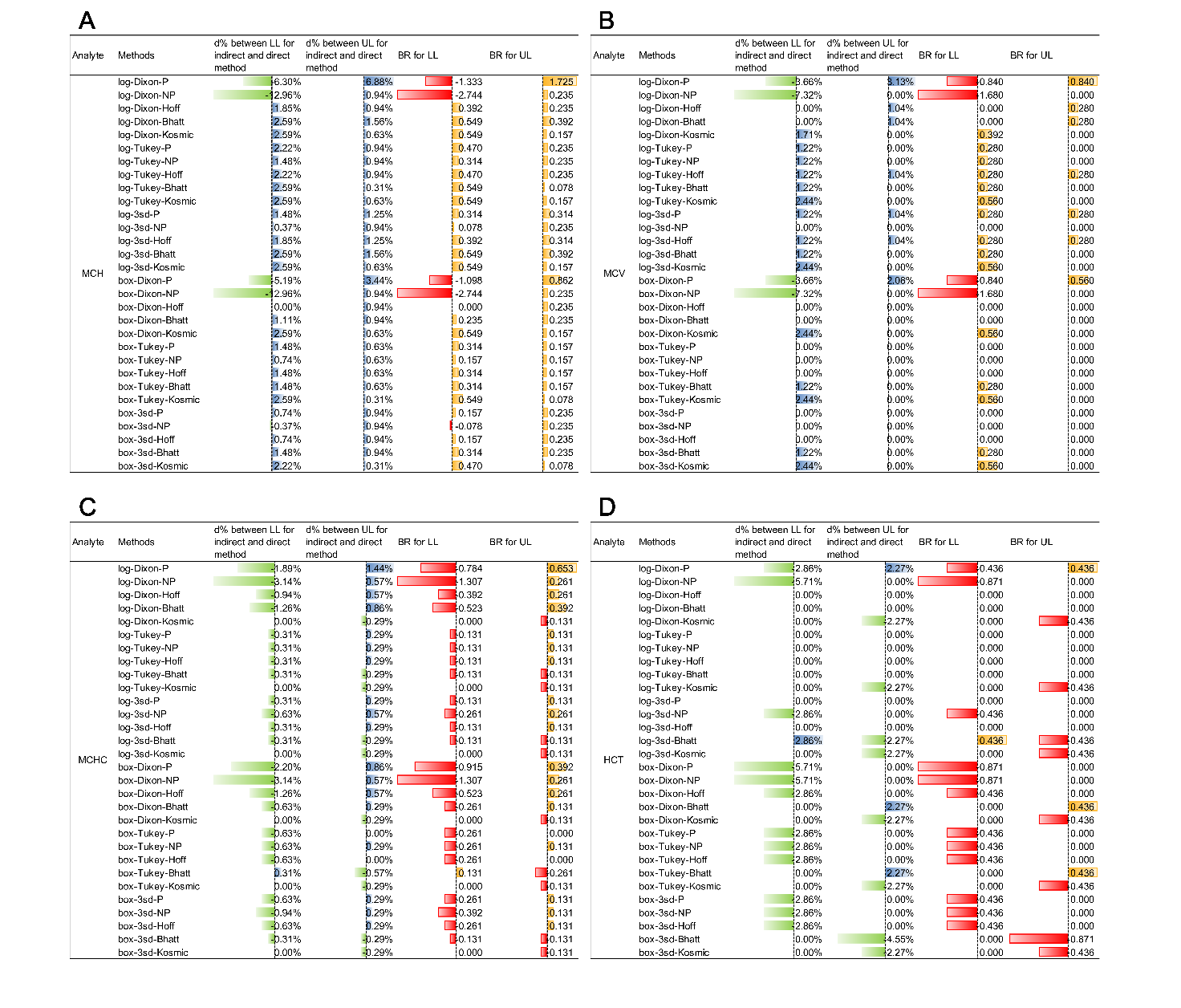
**
